# Long non-coding RNA CCDC183-AS1 acts AS a miR-589-5p sponge to promote the progression of hepatocellular carcinoma through regulating SKP1 expression

**DOI:** 10.1186/s13046-021-01861-6

**Published:** 2021-02-04

**Authors:** He Zhu, Hongwei Zhang, Youliang Pei, Zhibin Liao, Furong Liu, Chen Su, Yachong Liu, Renshun Dong, Jia Song, Xuewu Zhang, Yawei Fan, Huifang Liang, Bixiang Zhang, Xiaoping Chen

**Affiliations:** 1 Hepatic Surgery Center, Tongji Hospital, Tongji Medical College, Huazhong University of Science and Technology, Wuhan, Hubei P.R. China; 2 Hubei Key Laboratory of Hepato-Pancreato-Biliary Diseases, Wuhan, Hubei P.R. China; 3 Department of surgery, Tongji Hospital, Tongji Medical College, Huazhong University of Science and Technology, Wuhan, Hubei P.R. China; 4 Key Laboratory of Organ Transplantation, Ministry of Education, Wuhan, Hubei P.R. China

**Keywords:** Hepatocellular carcinoma, CCDC183-AS1, miR-589-5p, SKP1

## Abstract

**Background:**

Hepatocellular carcinoma (HCC) is a common type of malignant human cancer with high morbidity and poor prognosis, causing numerous deaths per year worldwide. Growing evidence has been demonstrated that long non-coding RNAs (lncRNAs) are closely associated with hepatocarcinogenesis and metastasis. However, the roles, functions, and working mechanisms of most lncRNAs in HCC remain poorly defined.

**Methods:**

Real-time quantitative polymerase chain reaction (qRT-PCR) was used to detect the expression level of CCDC183-AS1 in HCC tissues and cell lines. Cell proliferation, migration and invasion ability were evaluated by CCK-8 and transwell assay, respectively. Animal experiments were used to explore the role of CCDC183-AS1 and miR-589-5p in vivo. Bioinformatic analysis, dual-luciferase reporter assay and RNA immunoprecipitation (RIP) assay were performed to confirm the regulatory relationship between CCDC183-AS1, miR-589-5p and SKP1.

**Results:**

Significantly upregulated expression of CCDC183-AS1 was observed in both HCC tissues and cell lines. HCC patients with higher expression of CCDC183-AS1 had a poorer overall survival rate. Functionally, overexpression of CCDC183-AS1 markedly promoted HCC cell proliferation, migration and invasion in vitro and tumor growth and metastasis in vivo, whereas the downregulation of CCDC183-AS1 exerted opposite effects. MiR-589-5p inhibitor counteracted the proliferation, migration and invasion inhibitory effects induced by CCDC183-AS1 silencing. Mechanistically, CCDC183-AS1 acted as a ceRNA through sponging miR-589-5p to offset its inhibitory effect on the target gene SKP1, then promoted the tumorigenesis of HCC.

**Conclusions:**

CCDC183-AS1 functions as an oncogene to promote HCC progression through the CCDC183-AS1/miR-589-5p/SKP1 axis. Our study provided a novel potential therapeutic target for HCC patients.

**Supplementary Information:**

The online version contains supplementary material available at 10.1186/s13046-021-01861-6.

## Background

On a global basis, hepatocellular carcinoma (HCC) is one of the most lethal solid organ malignancies [1–3]. Hepatitis B virus (HBV) infection, chronic Hepatitis C virus (HCV) infection, aflatoxin exposure, alcohol abuse, obesity, smoking and type 2 diabetes are high risk factors associated with liver cancer [4–6]. Although the therapeutic effects of HCC have been improved, including surgical resection, liver transplantation and radiofrequency ablation, the overall prognosis for HCC patients remains unsatisfactory due to the high rate of postoperative recurrence and metastasis [7, 8]. Hence, further investigations of the molecular mechanisms underlying tumorigenesis and progression of HCC are urgently needed to develop more efficient therapeutic strategies [9].

Long non-coding RNA (lncRNA) is a kind of non-coding RNA molecules with more than 200 nucleotides in length [10, 11]. Recent studies have indicated that lncRNA participates in cancer development and progression, affects the proliferation, apoptosis and metastasis of tumor cells [12]. Of note, lncRNA is known to function as a competing endogenous RNA (ceRNA) to modulate target gene expression through absorbing miRNA [13, 14]. For example, lncRNA FAL1 promotes cell proliferation and migration by acting as a ceRNA of miR-1236 in hepatocellular carcinoma cells [15]; lncRNA n335586/miR-924/CKMT1A axis contributes to cell migration and invasion in hepatocellular carcinoma cells [16]. However, the molecular and cellular mechanisms of lncRNAs in HCC have not been fully elucidated.

To explore novel HCC-related lncRNAs, we analyzed the results of our RNA sequencing and public database. Among these differentially expressed lncRNAs, we selected and investigated CCDC183 antisense RNA 1 (CCDC183-AS1), a lncRNA located at human Chr 9q34.3 region. Here, we found that lncRNA CCDC183-AS1 was upregulated in HCC. Moreover, CCDC183-AS1 promoted HCC development and progression by the miR-589-5p/SKP1 axis, providing a new insight into the therapeutic strategy for HCC.

## Methods

### Patients and tissue specimens

HCC tissues and corresponding adjacent normal tissues were obtained from Hepatic Surgery Center, Tongji Hospital, Tongji Medical School, Huazhong University of Science and Technology (HUST, Wuhan, China) between 2012 and 2015 in accordance with the Helsinki Declaration. The study was approved by the Ethics Committee of Tongji Hospital and informed consents were obtained from all patients.

### The Cancer genome atlas (TCGA) data analysis and screening of differentially expressed lncRNAs

HCC RNAseq expression profile data were downloaded from the TCGA database (https://tcga-data.nci.nih.gov/tcga/) and analyzed as previously described [17]. HCC tissues and adjacent non-cancerous tissues were used for microarray analysis. The differentially expressed lncRNAs obtained from the TCGA database were combined with our own sequencing data, and a Venn diagram was generated.

### Cell lines and cell culture

The human liver cancer cell lines LM3 and 97H were obtained from the Liver Cancer Institute of Fudan University, Shanghai, China. HepG2, HLF, Hep3B, PLC/PRF/5 as well as normal-type hepatocyte HL7702 cells were purchased from China Center for Type Culture Collection (CCTCC, Wuhan, China). All cell lines were cultured in high-glucose Dulbecco’s modified Eagle’s medium (HyClone, USA) supplemented with 10% fetal bovine serum (Gibico, USA) at 37 °C in 5% CO_2_.

### Cell transfection

Cells were seeded on 6-well or 24-well plates at 50–70% confluence before transfection. SiCCDC183-AS1, miR-589-5p mimics, miR-589-5p inhibitor and the corresponding negative control were purchased from RiboBio (Guangzhou, China). Lipofectamine™ 3000 (Invitrogen, USA) was used as the transfection reagent according to the manufacturer’s protocol.

### Lentivirus production and transduction

The CCDC183-AS1, miR-589-5p and the corresponding negative control overexpression or knockdown recombinant lentiviruses were purchased from Genechem Co., Ltd. (Shanghai, China). All the lentivirus vectors were transduced according to the manufacturer’s instructions. Specifically, 2 × 10^5^ target cells were seeded into 6-well plates. The next day, 2 × 10^6^ virus and 40 μL infection reagent were added to the cells. The medium was replaced after 12 h, and cells were selected 48 h after the medium change. Infected cells were selected using puromycin (5 μg/mL) for 1 week.

### Fluorescence in situ hybridization (FISH)

FISH assay was performed to detect the location of CCDC183-AS1 in HCC cells. Cy3-labeled CCDC183-AS1 probes were ordered from RiboBio (Guangzhou, China). Hybridization steps were performed using Fluorescent In Situ Hybridization Kit (RiboBio, China) according to the manufacturer’s instructions. Confocal images were acquired on a laser scanning confocal microscope (LSM710, Carl Zeiss, Germany).

### Cell proliferation assay

Cell proliferation ability was examined using Cell Counting Kit-8 (CCK-8, Dojindo Crop, Japan). 1 × 10^3^ cells in 100 μL culture were plated in each well of a 96-well plate and incubated at 37 °C overnight for adherence. Every 24 h for a total of 96 h, each well was added with 10 μL of CCK-8 reagent and cells were further incubated for 1.5 h at 37 °C, the optical density (OD) was measured at 450 nm using a microplate reader (Bio-Tek Instruments, USA). These experiments were repeated for three times.

### Transwell migration and invasion assays

For the migration and invasion assays, according to the manufacturer’s protocol, HCC cells (2 × 10^4^ HLF cells, 3 × 10^4^ 97H cells and 5 × 10^4^ PLC/PRF/5 cells for migration assays; 4 × 10^4^ HLF cells, 5 × 10^4^ 97H cells and 1 × 10^5^ PLC/PRF/5 cells for invasion assays) were seeded in upper chambers with 100 μL of serum-free medium. The transwell chamber (8.0 μm; Corning, USA) was paved with 40 μL 1:4 mixture of matrigel (BD Biosciences, USA) and DMEM for invasion assays and paved without matrigel mix for migration assays. Meanwhile, DMEM Medium (700 μL) containing 10% fetal bovine serum was added to the lower chamber as a chemoattractant. After a 24 h incubation at 37 °C, the non-invading or non-migration cells on the upper membrane were removed mechanically. Cells on the lower surface of the membranes were fixed with 4% paraformaldehyde (Servicebio, China) for 15 min and stained with 0.1% crystal violet (Wuhan Promoter Biological Co., LTD) for 2 h. For visualization, the cells were photographed and counted from five random fields. Each experiment was repeated at least three times.

### RNA extraction and real-time quantitative polymerase chain reaction

The total RNAs of tissues and cell lines were extracted using TRIzol reagent (Takara, Japan) according to the manufacturer’s instructions. For lncRNA and mRNA, reverse transcriptions were performed using the HiScript® II Q RT SuperMix for qPCR (Vazyme Biotech Co., Ltd). For miRNA, reverse transcriptions were performed using the Mir-X miRNA First-Strand Synthesis Kit (Takara, Japan) and then cDNA amplification was performed using ChamQ Universal SYBR qPCR Master Mix (Vazyme Biotech Co., Ltd) following the manufacturer’s instructions with an CFX Connect™ real time system (Bio-Rad, USA). GAPDH and U6 were used as the endogenous control. Relative quantification of lncRNA, miRNA and mRNA expression were compared to endogenous control and analyzed using the comparative CT (2-ΔΔCT) method. All reactions were performed as triplicates. The primer sequences (Sangon Biotech, Shanghai, China) were available in Additional file: Supplementary Table 1.

### RNA immunoprecipitation

Magna RIP™ RNA-Binding Protein Immunoprecipitation Kit (Millipore, USA) was applied for RNA immunoprecipitation assay. Briefly, cells were lysed in complete RIP lysis buffer. Next, the cell extracts were incubated with magnetic beads conjugated with anti-Argonaute 2 (AGO2) (Abcam) or anti-IgG antibody (Abcam). Then, the beads were incubated with Proteinase K buffer to digest the protein. Finally, the immunoprecipitated RNA was isolated, purified and was further subjected to qRT-PCR analysis.

### Luciferase reporter assays

The sequences of CCDC183-AS1 and 3′-untranslated region (UTR) of the SKP1 and their corresponding mutation were designed, synthesized and inserted into luciferase reporter vector psiCHECK-2 (Promega, Madison, WI, USA), termed CCDC183-AS1-WT, CCDC183-AS1-MUT, SKP1–3’UTR-WT and SKP1–3’UTR-MUT, respectively. HLF and 97H cells (1 × 10^5^) were seeded in 24-well plates for 24 h before transfection. Subsequently, cells were co-transfected with 50 ng of the psiCHECK-2 vector and 50 nM of the miR-589-5p mimics or inhibitor using Lipofectamine 3000 (Invitrogen, USA). After 48 h of co-transfection, cell lysates were prepared using Passive Lysis Buffer (Promega), and luciferase activity was examined by Dual Luciferase Assay Kit (Promega, USA) in line with the manufacturer’s protocol. The relative luciferase activity of each sample was normalized to firefly luciferase activity. Experiments were repeated three times.

### Western blot (WB)

For Western blot assay, cells were lysed in RIPA buffer supplemented with protease and phosphatase inhibitor cocktail (MedChemExpress, USA) on ice for 30 min. The protein concentrations were quantified by BCA assay. Subsequently, equal amounts of protein were extracted by 10% SDS-PAGE and transferred onto Immobilon®-P transfer membranes (Millipore, USA). Then, the membranes were blocked in 5% skim milk at 37 °C for 1 h and incubated with primary antibodies anti-SKP1 (1:1000, Cell Signaling Technology, USA), anti-cyclin D1 (1:1000, Cell Signaling Technology, USA), anti-p21 (1:1000, Cell Signaling Technology, USA), anti-N-cadherin (1:1000, BD Biosciences, USA), anti-occludin (1:1000, Boster, BioEngineering Company, Wuhan, China) and anti-GAPDH (1:5000, Proteintech, China) at 4 °C overnight. Next, the prepared membranes were incubated with secondary antibody (1:5000, Aksomics, China) at 37 °C for 1 h. Finally, the blots were visualized by ECL chemiluminescent reagent (Bio-Rad, USA). Western blot band intensity was analyzed by Image Lab™ 4.0 software and GAPDH was used as a loading control to normalize the amount of protein.

### Immunohistochemistry (IHC) staining

Tumor tissues were fixed in 4% paraformaldehyde solution, embedded in paraffin and sectioned. Sections were first baked, deparaffinized in xylene and rehydrated in graded ethanol. Following washes in PBS, the samples were heated for antigen retrieval in boiling 0.01 mol/L citrate buffer (pH 6.0) for 15 min. The sections were then treated with 3% hydrogen peroxide in methanol for 10 min at room temperature. To prevent nonspecific antigen binding, the slides were blocked for 1 h at room temperature by 5% BSA and then incubated with the primary antibodies anti-SKP1 (1:200, Proteintech, China), anti-Ki67 (1:200, Abcam, USA) at 4 °C overnight. After rinses in PBS, sections were incubated for 1 h at room temperature with biotinylated anti-IgG secondary antibody. Subsequently, horseradish peroxidase-labeled streptavidin was added to the slides and incubated for 15 min. Lastly, DAB was used for chromogenic staining and counterstaining was performed with hematoxylin.

### Animal experiments

All animal care and experiments were carried out in accordance with the National Institutes of Health Guidelines for the Care and Use of Laboratory Animals and approved by the Ethics Committee of Tongji Hospital, affiliated to Tongji Medical College, HUST. Male BALB/c nude mice (4 weeks old) were purchased from Vital River Laboratory Animal Technology Co. Ltd. (Beijing, China). To generate the subcutaneous tumor model in nude mice, 1 × 10^6^ cells in 100 μL serum free DMEM were injected into the axillary region of mice. After 4 weeks, the mice were sacrificed and the tumor tissues were detected for tumor weight, volume, qRT-PCR, WB and IHC staining. For the orthotopic model, 1 × 10^6^ cells in 30 μL serum free DMEM were injected into the left hepatic lobe of nude mice. Thirty days later, the lungs and liver were removed and underwent HE staining.

### Statistical analysis

Statistical analyses were performed using GraphPad Prism 6.0 or SPSS 21.0 software. Data were compared using two-tailed Student’s t-test or one-way ANOVA test. Pearson correlation coefficient was used to analyze the linear correlations. Data are presented as mean ± SD. A *p* value of < 0.05 was considered statistically significant: ^*^ *p* < 0.05, ^**^ *p* < 0.01 and ^***^ *p* < 0.001; ns = not significant.

## Results

### CCDC183-AS1 expression was elevated in HCC

**Fig. 1:**
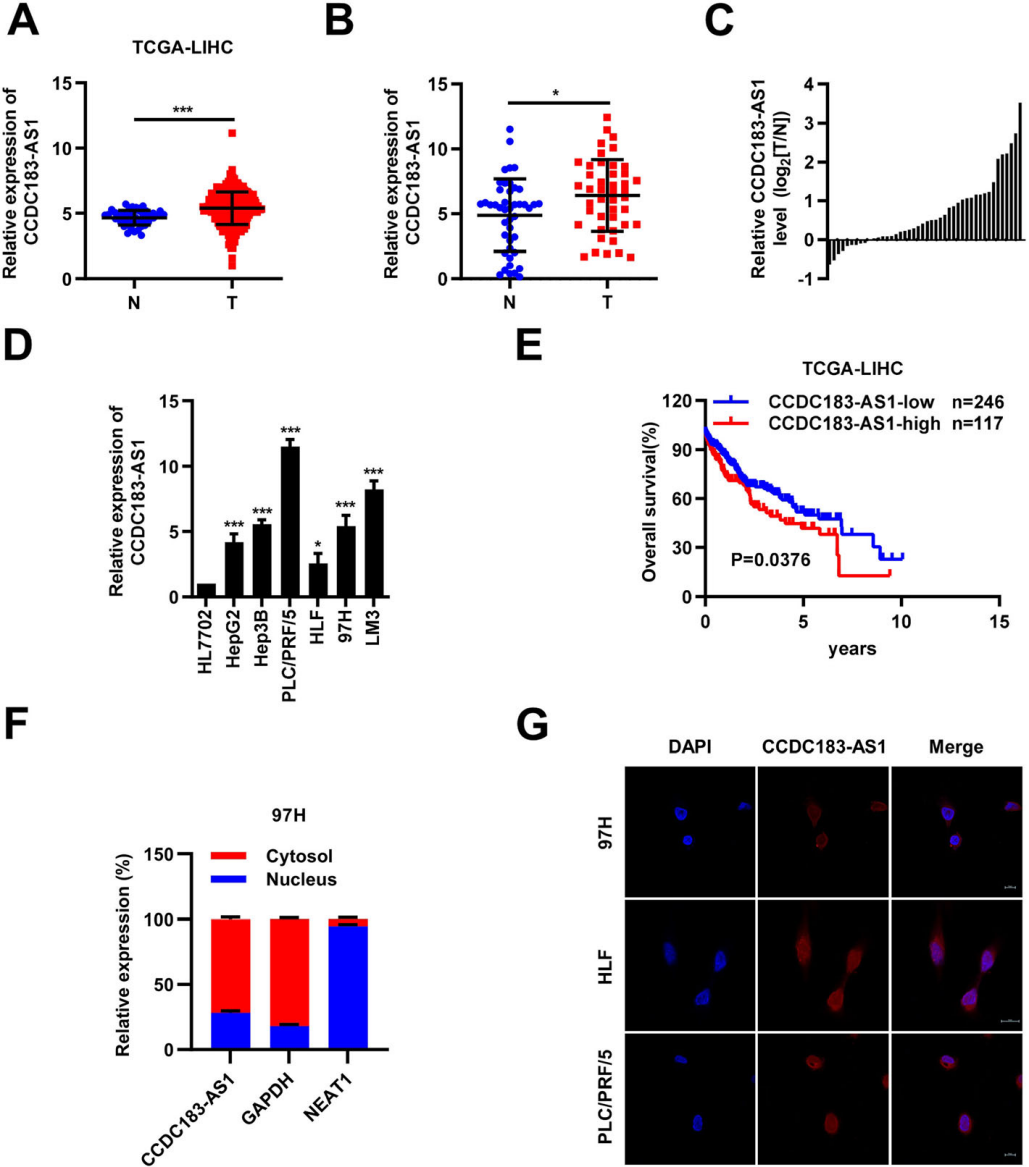
CCDC183-AS1 expression was elevated in HCC. **a**. Bioinformatic analysis of the expression of CCDC183-AS1 using TCGA database. **b**. qRT-PCR analysis of CCDC183-AS1 expression in HCC tissues and matched adjacent normal tissues(*n*=44). **c**. The relative expression of CCDC183-AS1 in 44 paired tissues [log_2_(T/N)]. **d**. Relative expression of CCDC183-AS1 in HCC cell lines was determined by qRT-PCR. **e**. The overall survival of HCC patients from the TCGA database with high and low CCDC183-AS1 expression. **f**. The expression level of CCDC183-AS1 in the subcellular fractions of 97H cells was detected by qRT-PCR. NEAT1 and GAPDH were used as nuclear and cytoplasmic markers, respectively. **g**. FISH assay analysis for the location of CCDC183-AS1 in HCC cells. Data are presented as mean ± SD. ^*^p < 0.05, ^**^p < 0.01, ^***^p < 0.001; ns, no significance. N, normal tissue; T, tumor tissue

A multitude of studies showed that lncRNAs played pivotal roles in cancer progression [18, 19]. In light of growing interest in lncRNA and their functions, we decided to investigate the role of lncRNAs in the progression of HCC. In this study, we mainly focused on upregulated lncRNAs in HCC. We first analyzed the HCC dataset from the TCGA database, 3488 upregulated lncRNAs and 594 downregulated lncRNAs were discovered (Supplementary Table 2). Meanwhile, we examined the results of our RNA sequencing and found 170 upregulated lncRNAs in HCC tissues relative to normal tissues (Supplementary Table 3). 15 lncRNAs were obtained by taking the intersection of 2 up-regulated lncRNA datasets mentioned above. We selected a novel lncRNA CCDC183-AS1, which had not been previously reported, for further investigation. We examined CCDC183-AS1 expression in TCGA database and found that it was significantly upregulated in HCC tissues compared to that in normal liver tissues (Fig. 1a). We further used qRT–PCR to determine the expression level of CCDC183-AS1 in 44 pairs of HCC and adjacent non-cancerous tissues. Our results showed that CCDC183-AS1 expression was markedly increased in HCC tissues compared with that in adjacent non-tumor tissues (Fig. 1b-c). Next, the expression levels of CCDC183-AS1 were measured in the HepG2, Hep3B, PLC/PRF/5, HLF, 97H and LM3 cell lines relative to normal human hepatocyte HL7702. Strikingly, elevated CCDC183-AS1 mRNA level was observed in HCC cell lines (Fig. 1d). Subsequently, we generated Kaplan–Meier curves of overall survival for TCGA cohort, and found that the patients with higher CCDC183-AS1 expression had poorer prognosis than those with low CCDC183-AS1 expression (Fig. 1e). The nuclear-cytoplasmic RNA fractionation and FISH assay were used to analyze the subcellular localization of CCDC183-AS1 in the HCC cells, and the results indicated that CCDC183-AS1 mostly located in the cytoplasm (Fig. 1f-g and Supplementary Fig. 1A-1B). These data above suggested that CCDC183-AS1 was upregulated in HCC and might be involved in HCC progression.

### CCDC183-AS1 promoted HCC cell proliferation and metastasis in vitro and in vivo

**Fig. 2:**
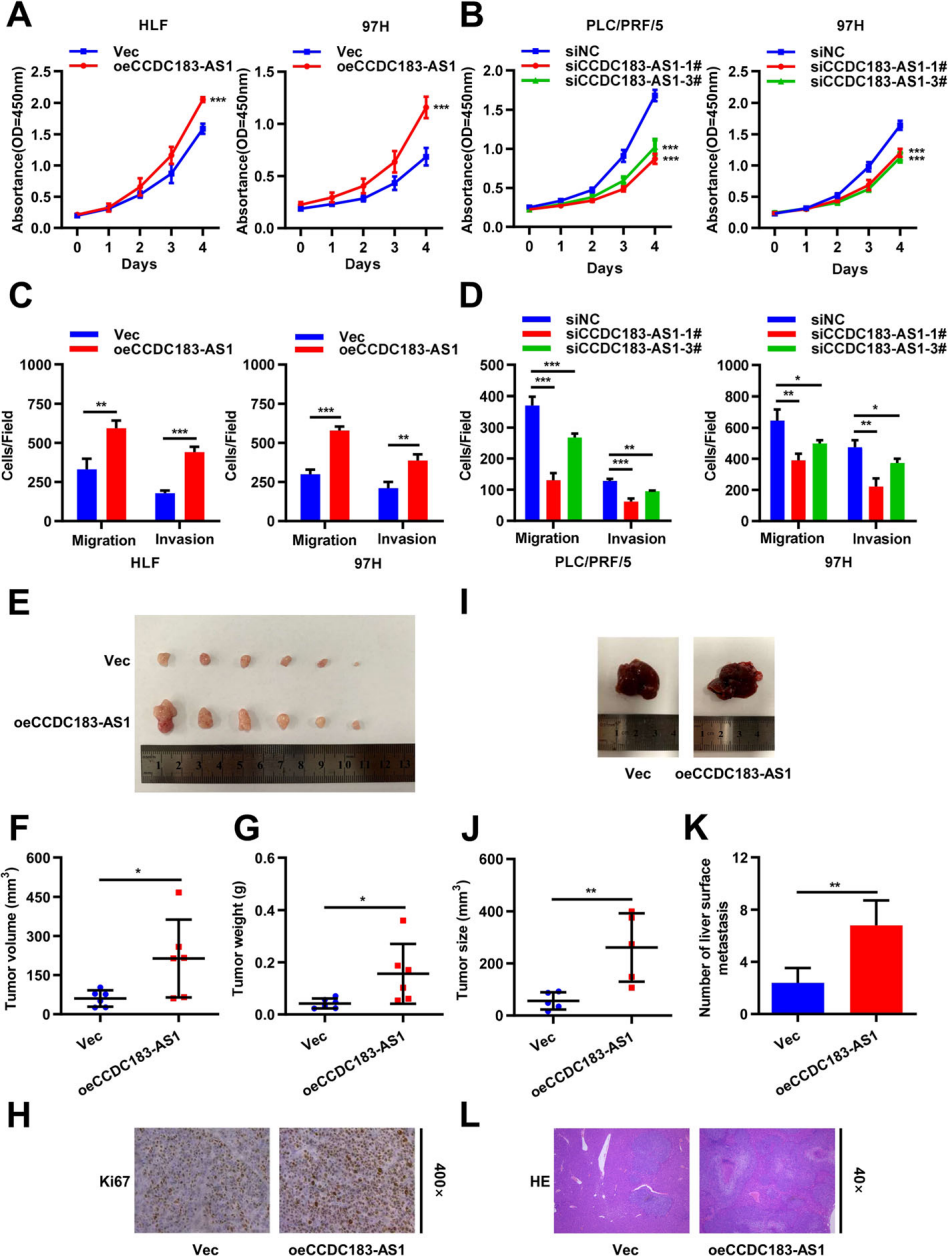
CCDC183-AS1 promoted HCC cell proliferation and metastasis in vitro and in vivo. **a-b**. CCK-8 assays were used to evaluate HCC cells proliferation after CCDC183-AS1 overexpression (**a**) or knockdown (**b**). **c-d**. Transwell experiments were performed to analyze the cell migration and invasion in CCDC183-AS1 overexpressing (**c**) and knockdown (**d**) HCC cells. **e**. Image of subcutaneous tumor tissues in CCDC183-AS1-overexpressing group and control group. **f-g**. The volume (**f**) and weight (**g**) of subcutaneous tumor tissues were measured. **h**. Relative expression levels of Ki67 were observed in subcutaneous tumor tissues by IHC. **i**. In orthotopic model mice, representative images of the liver of CCDC183-AS1 overexpressing group and control group. **j**. The size of the largest visible liver nodule was measured. **k**. Quantifications of visible surface liver metastatic nodules. **l**. Representative images in hematoxylin-eosin (HE) staining of the liver metastasis of 97H cells. Data are presented as mean ± SD. ^*^p < 0.05, ^**^p < 0.01, ^***^p < 0.001; ns, no significance

To explore the biological functions of CCDC183-AS1 in HCC cells, the overexpression vector of CCDC183-AS1 and corresponding control were transfected into HLF and 97H cells. PLC/PRF/5 and 97H cells were transfected with control siRNA or siRNA against CCDC183-AS1. The effects of CCDC183-AS1 overexpressing and knocking down in HCC cell lines were measured by qRT-PCR (Supplementary Fig. 2A-2B). CCK-8 assays indicated that the upregulation of CCDC183-AS1 significantly enhanced the proliferation viability, whereas the downregulation of CCDC183-AS1 exerted opposite effects (Fig. 2a-b). Then, transwell assays were carried out to examine the effects of CCDC183-AS1 on migration and invasion of HCC cells. The results suggested that overexpression of CCDC183-AS1 contributed to cell migration and invasion, whereas knockdown of CCDC183-AS1 significantly inhibited cell migration and invasion ((Fig. 2c-d and Supplementary Fig. 2C-2D).

To further explore the effects of CCDC183-AS1 on tumor growth and liver metastasis in vivo, we first subcutaneously injected 97H cells with stable overexpression of CCDC183-AS1 and corresponding control into BALB/c nude mice. Compared with the control group, the CCDC183-AS1 overexpression group remarkably increased the tumor volume and weight (Fig. 2e-g). IHC assays showed that upregulation of CCDC183-AS1 enhanced the expression of Ki-67 in xenograft tumor tissues (Fig. 2h). For the orthotopic model, 97H cells with stable overexpression of CCDC183-AS1 and corresponding control were injected into the left hepatic lobe of BALB/c nude mice. Compared with the control group, the CCDC183-AS1 overexpression group had more and larger liver metastatic nodules (Fig. 2i-l and Supplementary Fig. 2E). Taken together, these results indicated that CCDC183-AS1 promoted HCC cell proliferation and metastasis in vitro and in vivo.

### CCDC183-AS1 acted AS a ceRNA to sponge miR-589-5p in HCC cells

**Fig. 3:**
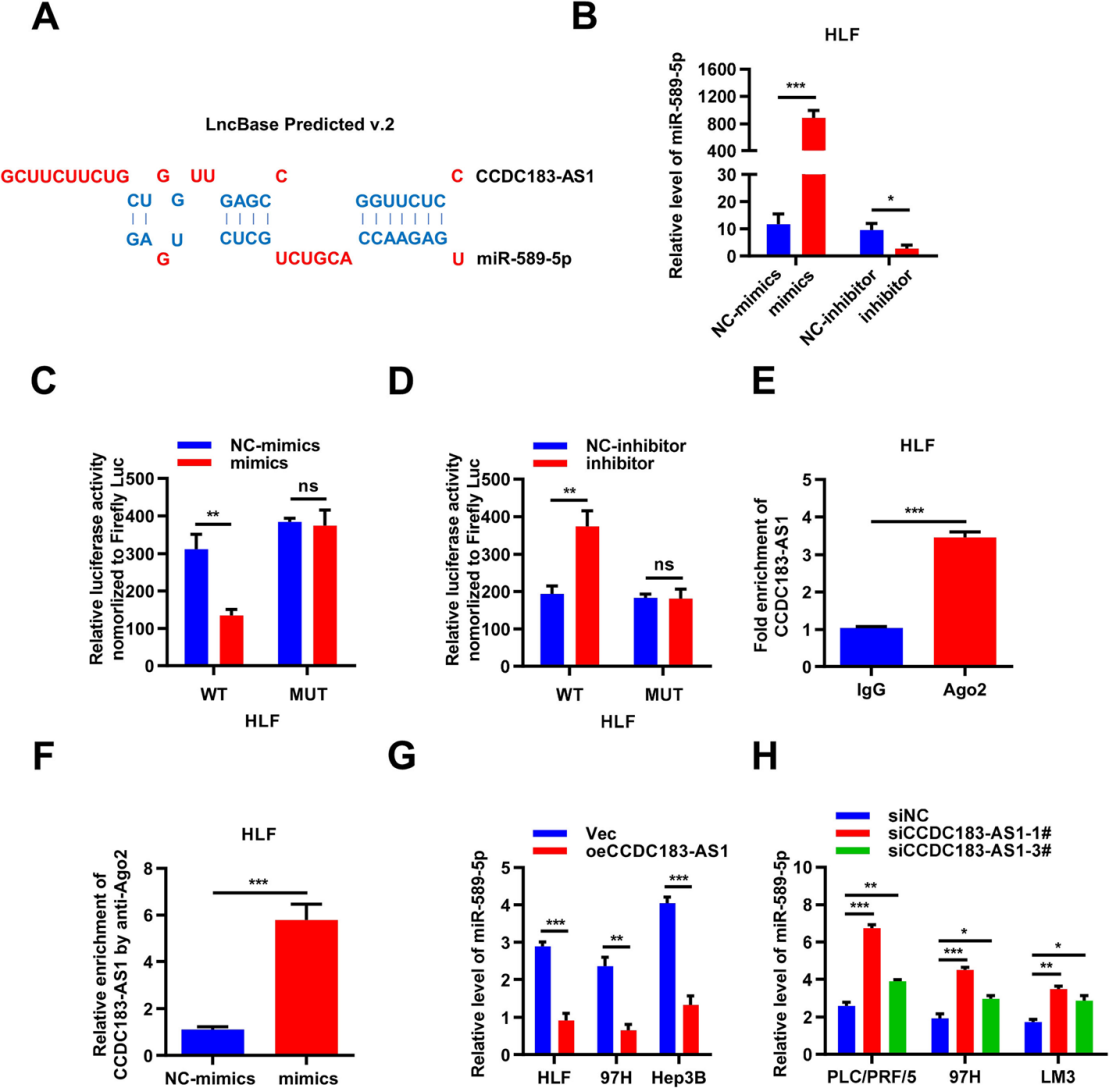
CCDC183-AS1 acted as a ceRNA to sponge miR-589-5p in HCC cells. **a**. Bioinformatic prediction (http://carolina.imis.athenainnovation.gr/diana_tools/web/index.php?r=lncbasev2%2Findex-predicted) of the potential binding site of miR-589-5p and CCDC183-AS1. **b**. Relative expression levels of miR-589-5p were evaluated by qRT-PCR in HLF cells transfected with the miR-589-5p mimics or inhibitor, respectively. **c-d**. The relative luciferase activities were detected in HLF cells after co-transfection with CCDC183-AS1-WT or CCDC183-AS1-MUT and mimics, inhibitor or NC, respectively. **e-f**. Anti-Ago2 RIP assay was executed in HLF cells after transfection with miR-589-5p mimics and NC, followed by qRT-PCR to detect CCDC183-AS1. **g-h**. qRT-PCR analysis of miR-589-5p expression in HCC cells after CCDC183-AS1 overexpression (**g**) or knockdown (**h**). Data are presented as mean ± SD. ^*^p < 0.05, ^**^p < 0.01, ^***^p < 0.001; ns, no significance

It is known that lncRNAs can modulate gene expression through numerous pathways, of which ceRNA appears to be a common mode of action for many lncRNAs [14, 20]. Given that CCDC183-AS1 mostly located in the cytoplasm of HCC cells, we further explored whether CCDC183-AS1 promoted the biological behavior of HCC by sponging miRNAs. Bioinformatics software such as DIANA Tools-LncBase Predicted v.2 [21] was used to predict the potential functional target miRNAs that could bind CCDC183-AS1. We found that CCDC183-AS1 might be a ceRNA for miR-589-5p (Fig. 3a). Then, we overexpressed miR-589-5p using RNA mimics and knocked down its expression using miR-589-5p inhibitors in HLF and 97H cells (Fig. 3b and Supplementary Fig. 3A). To confirm the bioinformatics prediction, dual-luciferase reporter assay was performed in HLF and 97H cells. We constructed the plasmids of wild-type (CCDC183-AS1-WT) and mutated (CCDC183-AS1-MUT) miR-589-5p binding site into psiCHECK-2 vector. We found that the relative luciferase activity in CCDC183-AS1-WT group was significantly decreased in miR-589-5p mimics group, while increased in miR-589-5p inhibitor group compared with that in NC group. And there was no significant difference among miR-589-5p mimics, inhibitor group and NC group in the relative luciferase activity of CCDC183-AS1-MUT group (Fig. 3c-d and Supplementary Fig. 3B-3C). The results indicated that an interaction might exist between CCDC183-AS1 and miR-589-5p. Subsequently, RIP assays were performed in an attempt to further verify the interaction between CCDC183-AS1 and miR-589-5p. The qRT-PCR results showed that CCDC183-AS1 was efficiently pulled down by anti-Ago2 and it was highly enriched in cells transfected with miR-589-5p mimics compared with that in control group (Fig. 3e-f). Furthermore, CCDC183-AS1 overexpression decreased miR-589-5p levels while CCDC183-AS1 silencing increased them (Fig. 3g-h). Collectively, these data demonstrated that CCDC183-AS1 acted as a sponge for miR-589-5p in HCC cells.

### miR-589-5p inhibited HCC cell proliferation and metastasis in vitro and in vivo

**Fig. 4:**
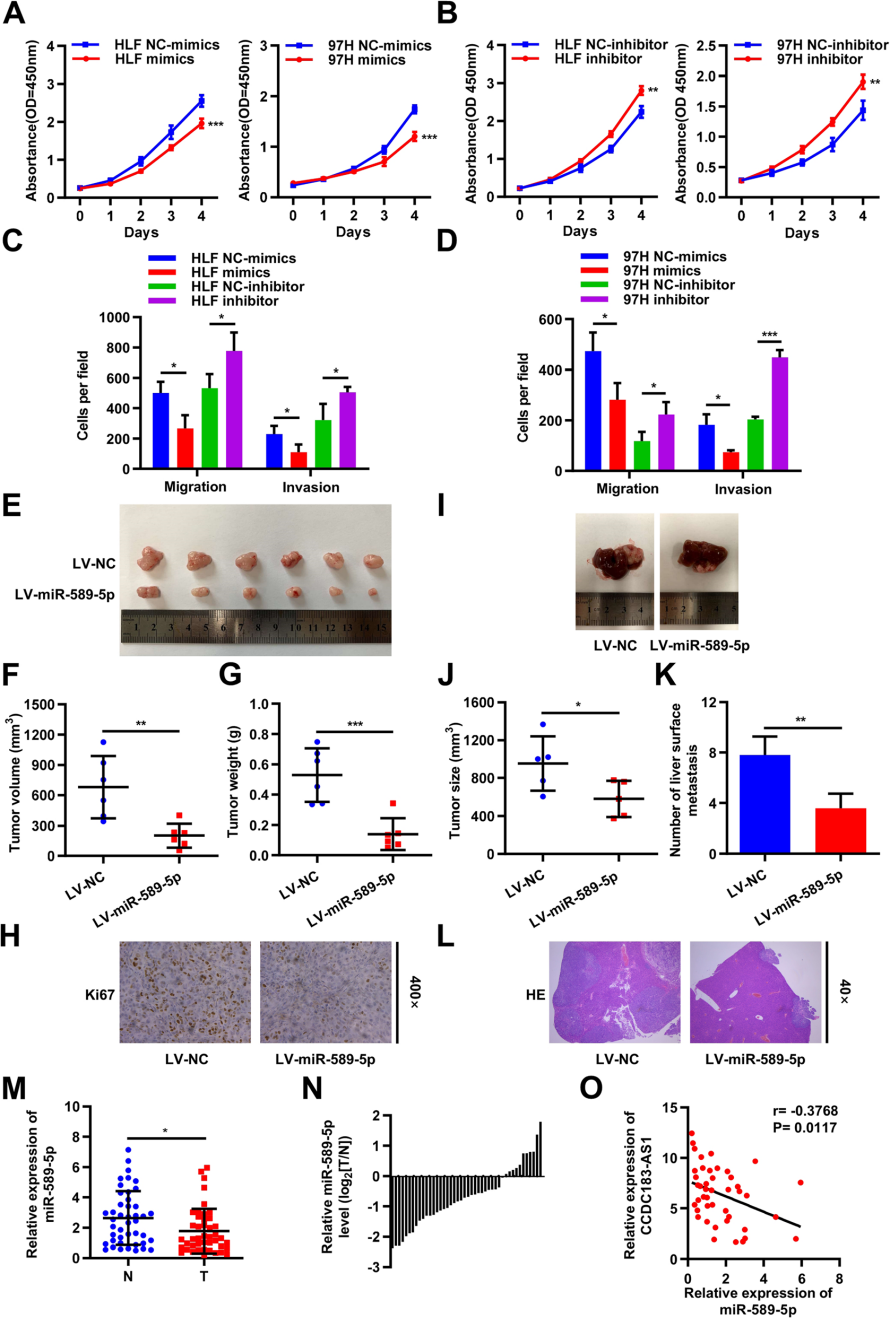
miR-589-5p inhibited HCC cell proliferation and metastasis in vitro and in vivo. **a-b**. Viability of HCC cells transfected with miR-589-5p mimics (**a**) or inhibitor (**b**) measured by CCK-8 assays. **c-d**. Cellular migratory and invasive capabilities were assessed by transwell assays in HLF and 97H cells transfected with miR-589-5p mimics (**c**) or inhibitor (**d**), respectively. **e**. Image of subcutaneous tumor tissues in miR-589-5p overexpressing group and control group. **f-g**. The volume (**f**) and weight (**g**) of subcutaneous tumor tissues were measured. **h**. Relative expression levels of Ki67 were observed in subcutaneous tumor tissues by IHC. **i**. In orthotopic model mice, representative images of the liver of miR-589-5p overexpressing group and control group. **j**. The size of the largest visible liver nodule was measured. **k**. Quantifications of visible surface liver metastatic nodules. **l**. Representative images in HE staining of the liver metastasis of HLF cells. **m**. qRT-PCR analysis of miR-589-5p expression in HCC tissues and matched adjacent normal tissues(*n*=44). **n**. The relative expression of miR-589-5p in 44 paired tissues [log_2_(T/N)]. **o**. miR-589-5p was negatively correlated with CCDC183-AS1 expression in 44 cases of HCC tissues measured by qRT-PCR. Data are presented as mean ± SD. ^*^p < 0.05, ^**^p < 0.01, ^***^p < 0.001; ns, no significance. N, normal tissue; T, tumor tissue

Given the above findings that CCDC183-AS1 might function by sponging miR-589-5p, we further investigated the biological functions of miR-589-5p. CCK-8 assays indicated that the upregulation of miR-589-5p significantly attenuated the proliferation viability, whereas decreased expression of miR-589-5p had exerted opposite effects (Fig. 4a-b). Transwell assays showed that the migratory and invasive capabilities of HLF and 97H cells were remarkably reduced by increased expression of miR-589-5p but significantly enhanced by decreased expression of miR-589-5p (Fig. 4c-d and Supplementary Fig. 4A-4B). Next, we utilized the subcutaneous xenograft model and orthotopic model to explore the role of miR-589-5p in vivo. We first used LV-miR-589-5p lentivirus vector to overexpress miR-589-5p in HLF cells (Supplementary Fig. 4C). Compared with the control group, miR-589-5p overexpression group possessed remarkably decreased tumor volume and weight (Fig. 4e-g). IHC analysis showed that the expression levels of Ki67 were markedly decreased in miR-589-5p-overexpressing tumors (Fig. 4h). For the orthotopic model, the miR-589-5p overexpression group had lower tumorigenic and metastasis potential in vivo compared with those in NC group (Fig. 4i-l and Supplementary Fig. 4D). We further used qRT–PCR to determine the expression level of miR-589-5p in 44 pairs of HCC and adjacent non-cancerous tissues. The results showed that miR-589-5p expression was significantly attenuated in HCC tissues compared with that in adjacent non-tumor tissues (Fig. 4m-n). Further, Pearson’s correlation analysis showed that the expression of miR-589-5p was negatively correlated with CCDC183-AS1 expression in 44 cases of HCC tissues (Fig. 4o). Taken together, these results suggested that miR-589-5p inhibited HCC cell proliferation and metastasis in vitro and in vivo.

### SKP1 is a direct target of miR-589-5p

**Fig. 5:**
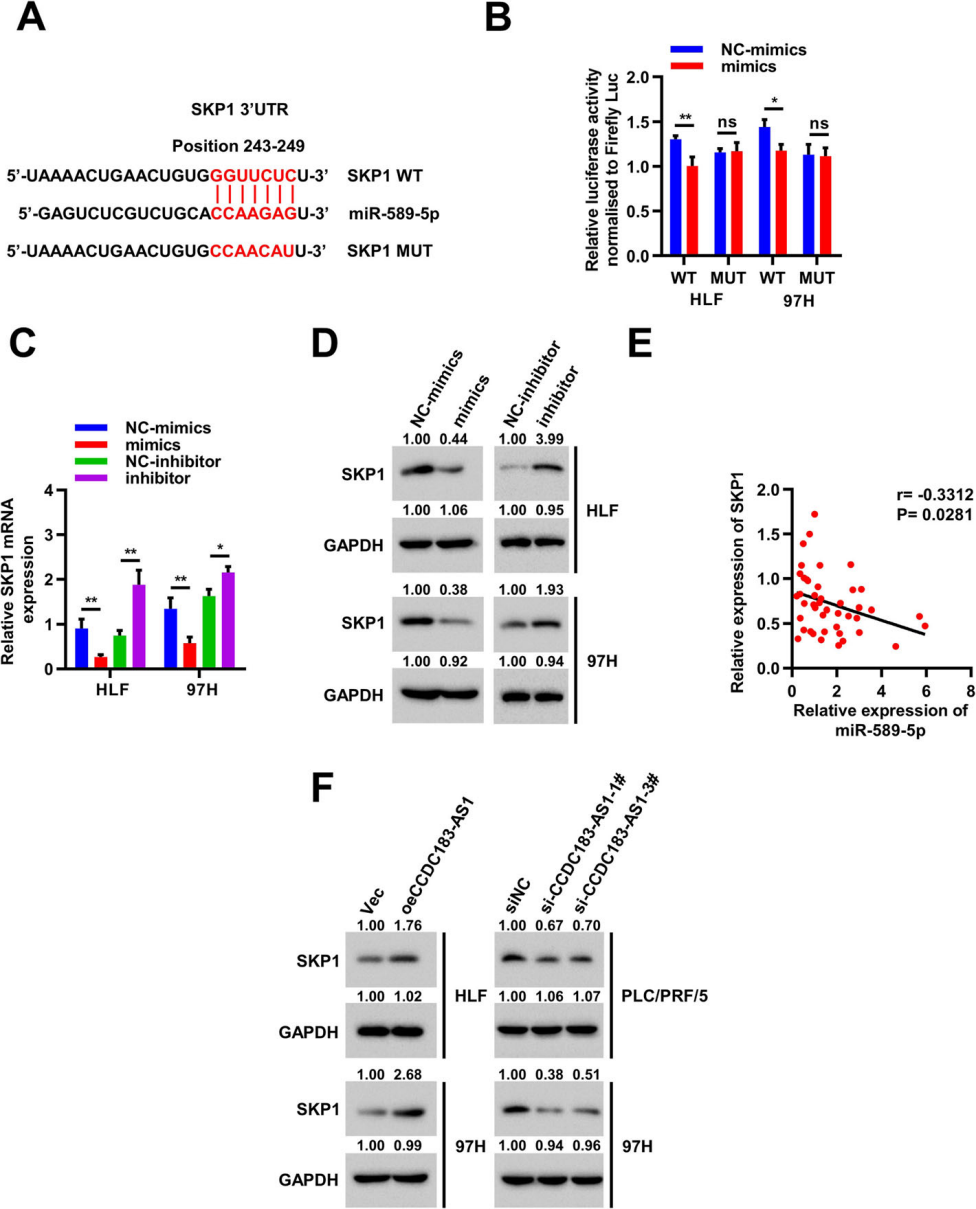
SKP1 is a direct target of miR-589-5p. **a**. Schematic diagram of miR-589-5p putative binding site in the WT and MUT 3’UTR of SKP1. **b**. The relative luciferase activities were detected in HLF and 97H cells after co-transfection with SKP1–3’UTR-WT or SKP1–3’UTR-MUT and mimics or NC, respectively. **c-d**. Relative mRNA (**c**) and protein (**d**) levels of SKP1 were evaluated by qRT-PCR and Western blot in HLF and 97H cells transfected with the miR-589-5p mimics or inhibitor, respectively. **e**. Correlation between miR-589-5p and SKP1 expression in paired HCC tissues (*n*=44). **f**. SKP1 protein levels in HCC cells after CCDC183-AS1 overexpression or knockdown. Data are presented as mean ± SD. ^*^p < 0.05, ^**^p < 0.01, ^***^p < 0.001; ns, no significance

To determine the target genes of miR-589-5p, we used five bioinformatics prediction tools (mirDIP, MicroT-CDS, TargetScan, miRWalk, miRDB) [22–26] to predict potential candidate targets (Supplementary Fig. 5A). After reviewing the relevant literature, we selected 10 genes most likely to be related to tumors from 41 candidate genes for qRT-PCR verification (Supplementary Fig. 5B-5F) and found that SKP1 might be a potential target gene of miR-589-5p. Next, the dual-luciferase reporter assay was used to verify whether miR-589–5p could combine with the SKP1 mRNA 3’UTR. And the sequence SKP1–3’UTR-MUT which destroyed the combing sites with miR-589-5p was constructed. The SKP1–3’UTR-WT and SKP1–3’UTR-MUT were cloned into luciferase reporter vector psiCHECK-2 (Fig. 5a) and then co-transfected with miR-589–5p mimics or NC into HLF and 97H cells, respectively. Results showed that the relative luciferase activity of SKP1–3’UTR-WT group significantly decreased in miR-589-5p mimics group compared with that in NC group, but these effects disappeared in the SKP1–3’UTR-MUT group (Fig. 5b). Moreover, qRT-PCR and immunoblot analyses further showed that miR-589-5p overexpression significantly decreased mRNA and protein expression of SKP1, whereas miR-589-5p knockdown had the opposite effects (Fig. 5c-d). In parallel, a negative correlation between miR-589-5p and SKP1 expression was observed in 44 cases of HCC tissues (Fig. 5e). We also found that CCDC183-AS1 overexpression markedly increased, while CCDC183-AS1 knockdown reduced the expression of SKP1 (Fig. 5f and Supplementary Fig. 5G). Overall, these results suggested that SKP1 is a direct target of miR-589-5p in HCC cells.

### SKP1 promoted HCC cell proliferation and metastasis

**Fig. 6:**
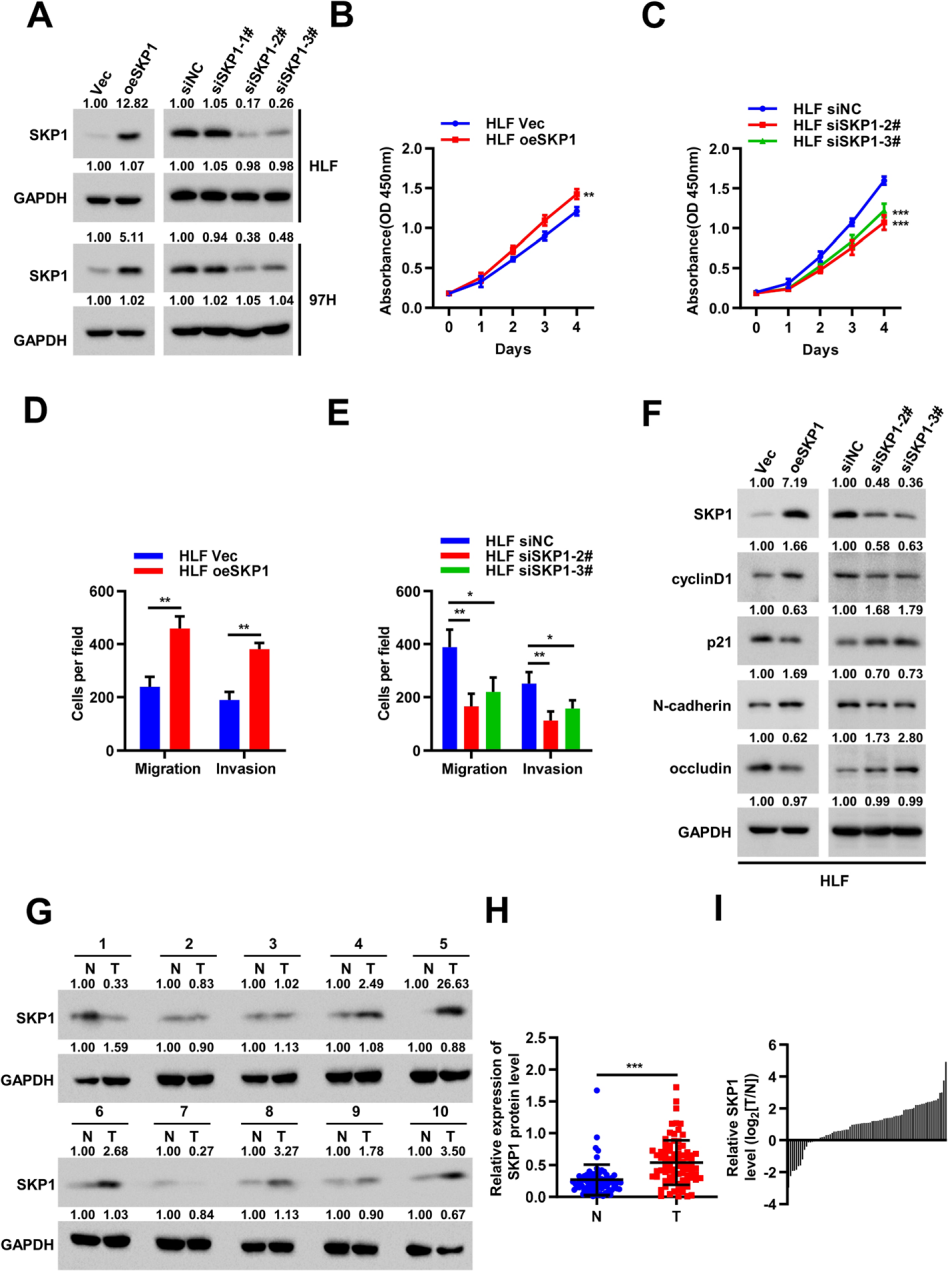
SKP1 promoted HCC cell proliferation and metastasis. **a**. The overexpression and knockdown efficiency of SKP1 were confirmed by Western blot. **b-c**. CCK-8 assays were used to evaluate HLF cells proliferation after SKP1 overexpression (**b**) or knockdown (**c**). **d-e**. Cellular migratory and invasive capabilities were assessed by transwell assays in HLF cells after SKP1 overexpression (**d**) or knockdown (**e**). **f**. Western blot analysis to determine expression level of the cell cycle and EMT relative marker in SKP1 overexpressed and knockdown HLF cells. **g**. The expression levels of SKP1 in HCC and adjacent non-cancer tissues were evaluated by Western blot(*n*=80). **h-i**. Statistical analysis of relative SKP1 levels in HCC tissues compared to normal tissue controls (n=80). Data are presented as mean ± SD. ^*^p < 0.05, ^**^p < 0.01, ^***^p < 0.001; ns, no significance. N, normal tissue; T, tumor tissue

As few studies had explored the role of SKP1 in HCC, we determined to investigate the biological functions of SKP1 in HCC cell lines. We first confirmed the overexpression and knockdown levels of SKP1 by Western blot analysis (Fig. 6a). CCK-8 assays indicated that the upregulation of SKP1 significantly enhanced the proliferation viability, while the opposite result was observed when SKP1 was downregulated (Fig. 6b-c and Supplementary Fig. 6A-6B). We then examined the migration and invasion ability of HCC cells using transwell assays. The results showed that SKP1 overexpression significantly promoted HLF and 97H cells migration and invasion, whereas SKP1 knockdown markedly inhibited these behaviors (Fig. 6d-e and Supplementary Fig. 6C-6F). Next, Western blot analysis was applied to measure the expression levels of cell cycle and Epithelial-mesenchymal transition (EMT) associated proteins, including cyclin D1, p21, N-cadherin, and occludin. The results demonstrated that cyclin D1, N-cadherin were upregulated, and p21, occludin were downregulated in CCDC183-AS1 overexpression HCC cells, while the expression levels of these indicators were reversed when CCDC183-AS1 was attenuated (Fig. 6f and Supplementary Fig. 6G). Furthermore, the expression levels of SKP1 in 80 pairs of HCC and adjacent non-cancer tissues were evaluated by Western blot. Results showed that SKP1 expression was markedly increased in HCC tissues compared with that in adjacent non-tumor tissues (Fig. 6g-i and Supplementary Fig. 6H). In summary, SKP1 promoted HCC cell proliferation and metastasis.

### CCDC183-AS1 promoted HCC progression through the CCDC183-AS1/miR-589-5p/SKP1 axis

**Fig. 7:**
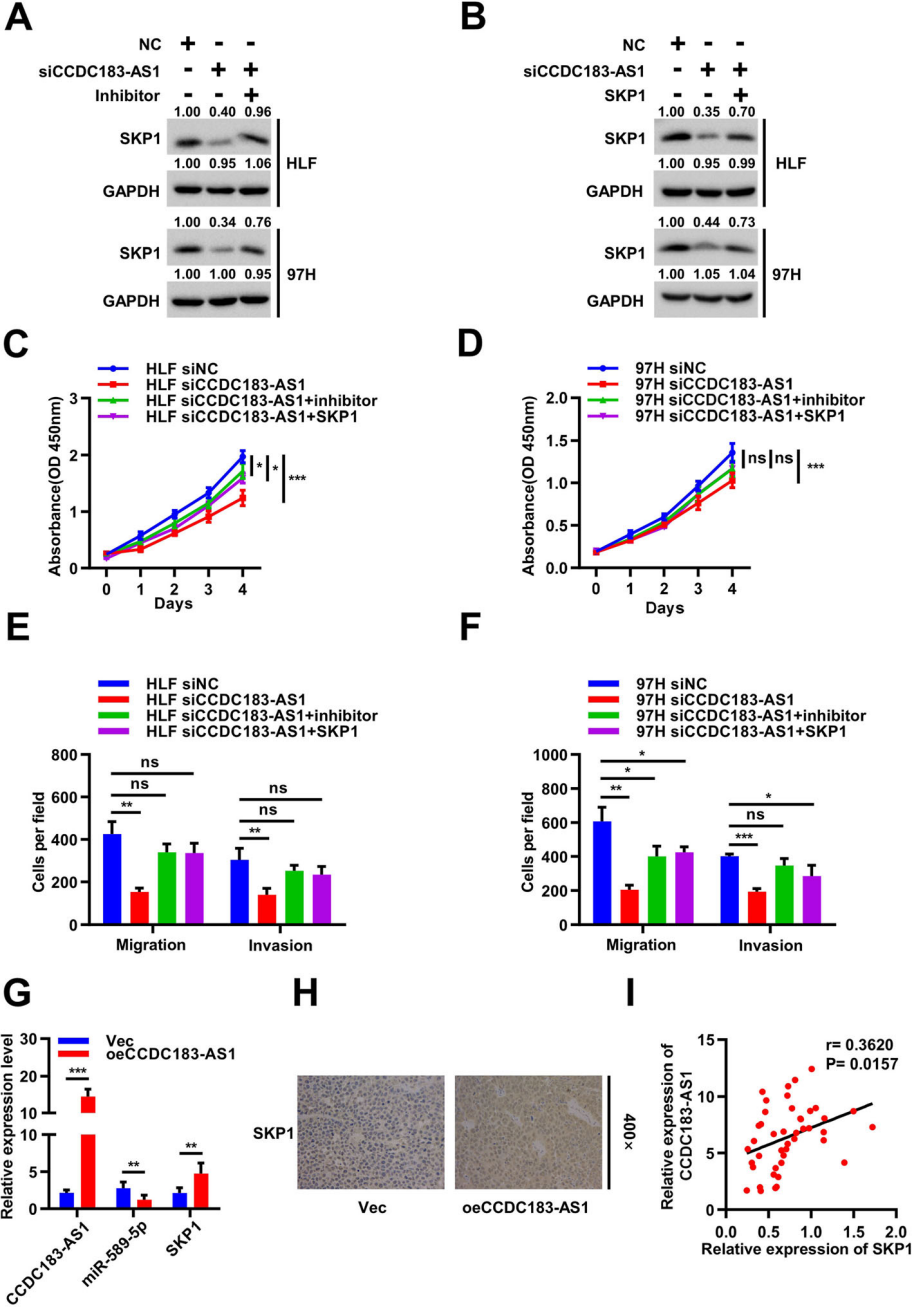
CCDC183-AS1 promotes HCC progression through the CCDC183-AS1/miR-589-5p/SKP1 axis. **a-b**. Relative protein levels of SKP1 were detected in HLF and 97H cells transfected with indicated NC, siCCDC183-AS1, miR-589-5p inhibitor or SKP1 using Western blot, respectively. **c-d**. CCK-8 assays were performed to determine the ability of proliferation in HLF and 97H cells transfected with indicated NC, siCCDC183-AS1, miR-589-5p inhibitor or SKP1, respectively. **e-f**. Cell migratory and invasive capabilities were assessed by transwell assays in HLF and 97H cells transfected with indicated NC, siCCDC183-AS1, miR-589-5p inhibitor or SKP1, respectively. **g**. Relative expression levels of CCDC183-AS1, miR-589-5p and SKP1 were measured in subcutaneous tumor tissues by qRT-PCR. **h**. Relative expression levels of SKP1 were observed in subcutaneous tumor tissues by IHC. **i**. Correlation between CCDC183-AS1 and SKP1 expression in paired HCC tissues (*n*=44). Data are presented as mean ± SD. ^*^p < 0.05, ^**^p < 0.01, ^***^p < 0.001; ns, no significance

To verify whether CCDC183-AS1 exerted its tumor promoting function through CCDC183-AS1/miR-589-5p/SKP1 axis, we performed rescue experiments using miR-589-5p inhibitor and SKP1 overexpression. Western blot assays showed that SKP1 protein levels in HLF and 97H cells were dramatically decreased after CCDC183-AS1 knockdown. Simultaneously, the effects caused by silencing CCDC183-AS1 were reversed by miR-589-5p inhibitor (Fig. 7a). Consistently, SKP1 overexpression greatly abolished the suppressive effects of CCDC183-AS1 knockdown (Fig. 7b). Next, we further sought to explore the biological functions of CCDC183-AS1 in HCC cells and found that they could also be reversed by miR-589-5p inhibitor or SKP1 overexpression. Results of CCK-8 and transwell assays demonstrated that miR-589-5p inhibitor reversed the proliferation, migration and invasion suppressive effects induced by CCDC183-AS1 knockdown in HLF and 97H cells. SKP1 overexpression yielded similar results (Fig. 7c-f and Supplementary Fig. 7A-7B). In addition, the expression levels of CCDC183-AS1, miR-589-5p and SKP1 were measured by qRT-PCR in xenograft tumors. The results showed that CCDC183-AS1 and SKP1 expression was increased, whereas miR-589-5p expression was decreased (Fig. 7g). IHC assays showed that upregulation of CCDC183-AS1 enhanced the expression of SKP1 in xenograft tumor tissues (Fig. 7h). Further analysis suggested that SKP1 protein expression was positively correlated with CCDC183-AS1 expression in HCC tissues (Fig. 7i). Taken together, these results indicated that CCDC183-AS1 served as a ceRNA for miR-589-5p to regulate SKP1 expression, thus leading to the progression and development of HCC.

## Discussion

Owing to recent advances in high-throughput sequencing, more and more lncRNAs have been annotated [19, 27]. Accumulating evidence has demonstrated that lncRNAs are involved in cancer progression [28]. Some lncRNAs were reported to function as oncogenes or tumor suppressors in a variety of human cancers, including HCC [29–31]. In addition to the well characterized lncRNAs, it remains necessary to further investigate potential essential lncRNAs in controlling HCC progression [32]. In this study, we first analyzed the results of our RNA sequencing and TCGA database about the differentially expressed lncRNAs in HCC and normal liver tissues. We identified a novel lncRNA CCDC183-AS1, which was obviously upregulated in HCC tissues and cell lines compared with that in adjacent non-cancerous tissues and normal human hepatocyte, respectively. Moreover, TCGA data showed that high expression of CCDC183-AS1 was significantly associated with poor overall survival. These findings above suggested that CCDC183-AS1 might be involved in HCC progression.

To our knowledge, no studies have yet analyzed the functions of CCDC183-AS1 up to date. We therefore investigated the biological functions of CCDC183-AS1 in HCC cells both in vitro and in vivo. The results indicated that CCDC183-AS1 overexpression significantly promoted cell proliferation, migration and invasion, whereas CCDC183-AS1 knockdown exerted opposite effects. Consistent with these findings, animal experiments also found that CCDC183-AS1 overexpression remarkably promoted tumor growth and metastasis. Taken together, these results suggest an oncogenic role of CCDC183-AS1 in HCC and highlight the need for further investigation to determine the molecular mechanisms of CCDC183-AS1 involving in HCC.

The nuclear-cytoplasmic RNA fractionation and FISH assay revealed that CCDC183-AS1 mostly located in the cytoplasm. Previous researches indicated that the cytoplasmic lncRNAs played critical roles in multiple molecular mechanisms, especially as ceRNAs [18, 33]. We speculated, therefore, that CCDC183-AS1 may function as ceRNA and participate in the carcinogenesis of HCC. We used bioinformatics software to predict the potential functional target miRNAs and found that CCDC183-AS1 might be a ceRNA for miR-589-5p. Dual-luciferase reporter assay and RIP assay confirmed that CCDC183-AS1 might directly bind to miR-589-5p. Moreover, CCDC183-AS1 overexpression decreased miR-589-5p levels while CCDC183-AS1 silencing increased them in HCC cells. HCC tissues from our cohort also showed an inverse correlation between CCDC183-AS1 and miR-589-5p expression. Collectively, these data demonstrated that CCDC183-AS1 acted as a sponge for miR-589-5p in HCC cells.

Current literature on the role of miR-589-5p in HCC remains controversial. Zhang et al. reported that miR-589-5p inhibits MAP 3 K8 and suppresses CD90+ cancer stem cells in hepatocellular carcinoma [34]. Zhu et al. found that miR-589-5p is downregulated in HCC tissues and functions as a tumor suppressor [35]. Conversely, some scholars hold the opposite opinion [36, 37]. Therefore, further studies are warranted to confirm the roles of miR-589-5p in HCC. From functional study in vitro and in vivo, we found that the upregulation of miR-589-5p significantly attenuated HCC cell proliferation and metastasis, while miR-589-5p knockdown displayed the opposite tendency. Meanwhile, miR-589-5p expression was significantly decreased in HCC tissues compared with that in adjacent non-tumor tissues. These results indicated that miR-589-5p might act as a tumor suppressor in HCC. Thus, we believe that CCDC183-AS1 plays carcinogenic effects in HCC by down-regulating the expression of miR-589-5p.

Next, we attempted to find the potential target genes of miR-589-5p by using bioinformatics analysis. The results of qRT-PCR and dual-luciferase reporter assay confirmed that SKP1 was a direct target of miR-589-5p. Moreover, we discovered that miR-589-5p overexpression significantly decreased mRNA and protein expression of SKP1 and a negative correlation between miR-589-5p and SKP1 expression was observed in HCC tissues, suggesting that miR-589-5p was an important negative regulator of the SKP1. SKP1 is a part of the SKP1–Cullin–F-box (SCF) ubiquitin E3 ligase and plays an important role in ubiquitin-mediated degradation of some cell-cycle regulatory proteins [38–40]. For example, SCF-SKP2 complex functions largely as an oncogene to mediate the protein degradation of CDK inhibitors, such as p21, p27, and p57 [39, 41, 42]. In our study, we investigated the biological functions of SKP1 in HCC cell lines and found that SKP1 promoted HCC cell proliferation and metastasis. Furthermore, we also found that CCDC183-AS1 overexpression markedly increased, while CCDC183-AS1 silencing reduced SKP1 protein levels. Taken together, we believe that CCDC183-AS1 may function as a ceRNA through sponging miR-589-5p to offset its inhibitory effect on the target gene SKP1 in HCC.

Finally, a negative correlation between CCDC183-AS1 and miR-589-5p expression and a positive correlation between CCDC183-AS1 and SKP1 expression were observed in xenograft tumor and HCC tissues. Importantly, rescue experiments further confirmed that miR-589-5p inhibitor reversed the proliferation, migration and invasion inhibitory effects induced by CCDC183-AS1 silencing. Similarly, SKP1 partially reversed the knockdown effect of CCDC183-AS1, suggesting that the suppressive effect of CCDC183-AS1 silencing on HCC cells was dependent on the inhibition of SKP1 expression.

## Conclusion

In summary, our findings identified a novel lncRNA CCDC183-AS1, which was elevated in HCC tissues and cell lines and functioned as an oncogene. HCC patients with higher expression of CCDC183-AS1 had a poorer overall survival rate. Functional analysis suggested that CCDC183-AS1 overexpression promoted HCC cell proliferation and metastasis. Mechanistically, CCDC183-AS1 acted as a miR-589-5p sponge to promote HCC progression through regulating SKP1 expression. Our study provided a novel potential therapeutic target for HCC patients.

## Supplementary Information

**Additional file 1: Supplementary Figure S1.** CCDC183-AS1 expression was elevated in HCC. a-b. The expression level of CCDC183-AS1 in the subcellular fractions of HLF cells (a) and PLC/PRF/5 cells (b) were detected by qRT-PCR. NEAT1 and GAPDH were used as nuclear and cytoplasmic markers, respectively.

**Additional file 2: Supplementary Figure S2.** CCDC183-AS1 promoted HCC cell proliferation and metastasis in vitro and in vivo. a-b. The overexpression (a) and knockdown (b) efficiency of CCDC183-AS1 were examined by qRT-PCR. c-d. Representative images of transwell assays in CCDC183-AS1 overexpressed (c) or knockdown (d) HCC cells. e. Representative images of the liver of CCDC183-AS1 overexpressing group and control group. Data are presented as mean ± SD. ^*^p < 0.05, ^**^p < 0.01, ^***^p < 0.001; ns, no significance.

**Additional file 3: Supplementary Figure S3.** CCDC183-AS1 acted as a ceRNA to sponge miR-589-5p in HCC cells. a. Relative expression levels of miR-589-5p were evaluated by qRT-PCR in 97H cells transfected with the miR-589-5p mimics or inhibitor, respectively. b-c. The relative luciferase activities were detected in 97H cells after co-transfection with CCDC183-AS1-WT or CCDC183-AS1-MUT and mimics, inhibitor or NC, respectively. Data are presented as mean ± SD. ^*^p < 0.05, ^**^p < 0.01, ^***^p < 0.001; ns, no significance.

**Additional file 4: Supplementary Figure S4.** miR-589-5p inhibited HCC cell proliferation and metastasis in vitro and in vivo. a-b. Representative images of transwell assays in HLF (a) and 97H (b) cells transfected with miR-589-5p mimics or inhibitor, respectively. c. The expression levels of miR-589-5p were examined by qRT-PCR in HLF and 97H cells with stable miR-589-5p overexpression or knockdown. d. Representative images of the liver of miR-589-5p overexpressing group and control group. Data are presented as mean ± SD. ^*^p < 0.05, ^**^p < 0.01, ^***^p < 0.001; ns, no significance.

**Additional file 5: Supplementary Figure S5.** SKP1 is a direct target of miR-589-5p. a. Venn diagrams showing the number of target genes of miR-589-5p predicted by mirDIP, MicroT-CDS, TargetScan, miRWalk and miRDB. b-d. Expression levels of candidate genes were measured by qRT-PCR after CCDC183-AS1 overexpression (b-c) or knockdown (d). e-f. qRT-PCR analysis of the expression of candidate genes in HLF (e) and 97H (f) cells when treated with miR-589-5p mimics or inhibitor. g. SKP1 protein levels in HLF cells after CCDC183-AS1 knockdown. Data are presented as mean ± SD. ^*^p < 0.05, ^**^p < 0.01, ^***^p < 0.001; ns, no significance.

**Additional file 6: Supplementary Figure S6.** SKP1 promoted HCC cell proliferation and metastasis. a-b. CCK-8 assays were used to evaluate 97H cells proliferation after SKP1 overexpression (a) or knockdown (b). c-d. Cellular migratory and invasive capabilities were assessed by transwell assays in 97H cells after SKP1 overexpression (c) or knockdown (d). e-f. Representative images of transwell assays in HLF and 97H cells after SKP1 overexpression (e) or knockdown (f). g. Western blot analysis to determine expression level of the cell cycle and EMT relative marker in SKP1 overexpressed and knockdown 97H cells. h. The expression levels of SKP1 in HCC and adjacent non-cancer tissues were evaluated by Western blot(*n*=80). Data are presented as mean ± SD. ^*^p < 0.05, ^**^p < 0.01, ^***^p < 0.001; ns, no significance.

**Additional file 7: Supplementary Figure S7.** CCDC183-AS1 promotes HCC progression through the CCDC183-AS1/miR-589-5p/SKP1 axis. a-b. Representative images of transwell assays in HLF (a) and 97H (b) cells transfected with indicated NC, siCCDC183-AS1, miR-589-5p inhibitor or SKP1, respectively.

**Additional file 8.**

**Additional file 9.**

**Additional file 10.**

## Data Availability

All the data and materials supporting the conclusions were included in the main paper.

## Abbreviations

lncRNA: Long non-coding RNA
HCC: Hepatocellular carcinoma
CCDC183-AS1: CCDC183 antisense RNA 1
HBV: Hepatitis B virus
HCV: Hepatitis C virus
ceRNA: Competing endogenous RNA
miRNA: MicroRNA
qRT-PCR: Real-time quantitative polymerase chain reaction
TCGA: The Cancer Genome Atlas
RIP: RNA immunoprecipitation
3’UTR: 3′-untranslated region
EMT: Epithelial-mesenchymal transition
CCK-8: Cell Counting Kit-8
NC: Negative control
oe: Overexpressed
OD: Optical density
LIHC: Liver hepatocellular carcinoma
FISH: Fluorescence in situ hybridization
IHC: Immunohistochemistry
HE: Hematoxylin-eosin
WB: Western blot
Ago2: Argonaute 2
