# Supplementary figures and images for: Long non-coding RNA CCDC183-AS1 acts AS a miR-589-5p sponge to promote the progression of hepatocellular carcinoma through regulating SKP1 expression
Source: J Exp Clin Cancer Res. 2021 Feb 4;40:57. doi: 10.1186/s13046-021-01861-6 (PMC7863448; doi:10.1186/s13046-021-01861-6)

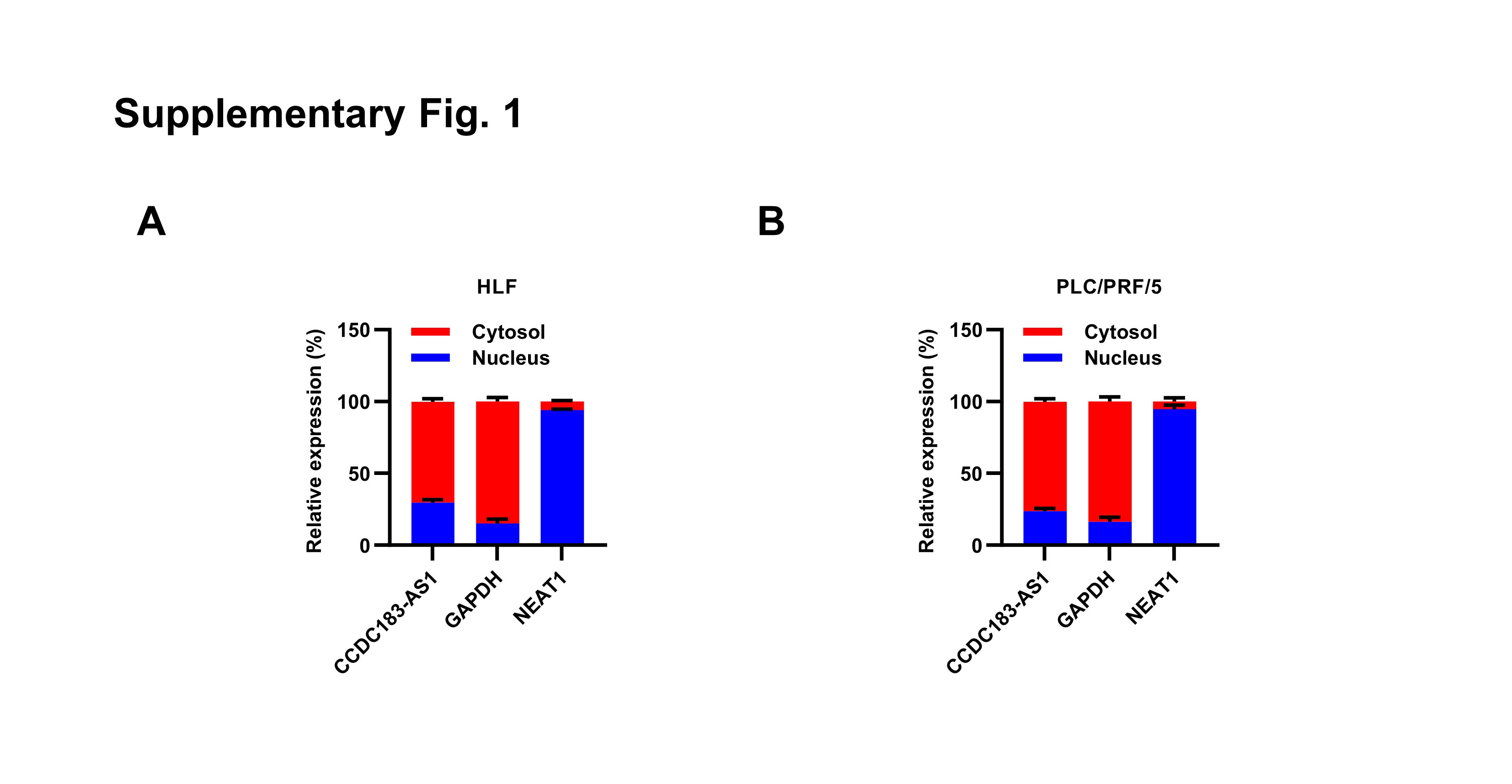

Supplement: Supplementary file 1 — Additional file 1: Supplementary Figure S1. CCDC183-AS1 expression was elevated in HCC. a-b. The expression level of CCDC183-AS1 in the subcellular fractions of HLF cells (a) and PLC/PRF/5 cells (b) were detected by qRT-PCR. NEAT1 and GAPDH were used as nuclear and cytoplasmic markers, respectively. [file 13046_2021_1861_MOESM1_ESM.jpg]

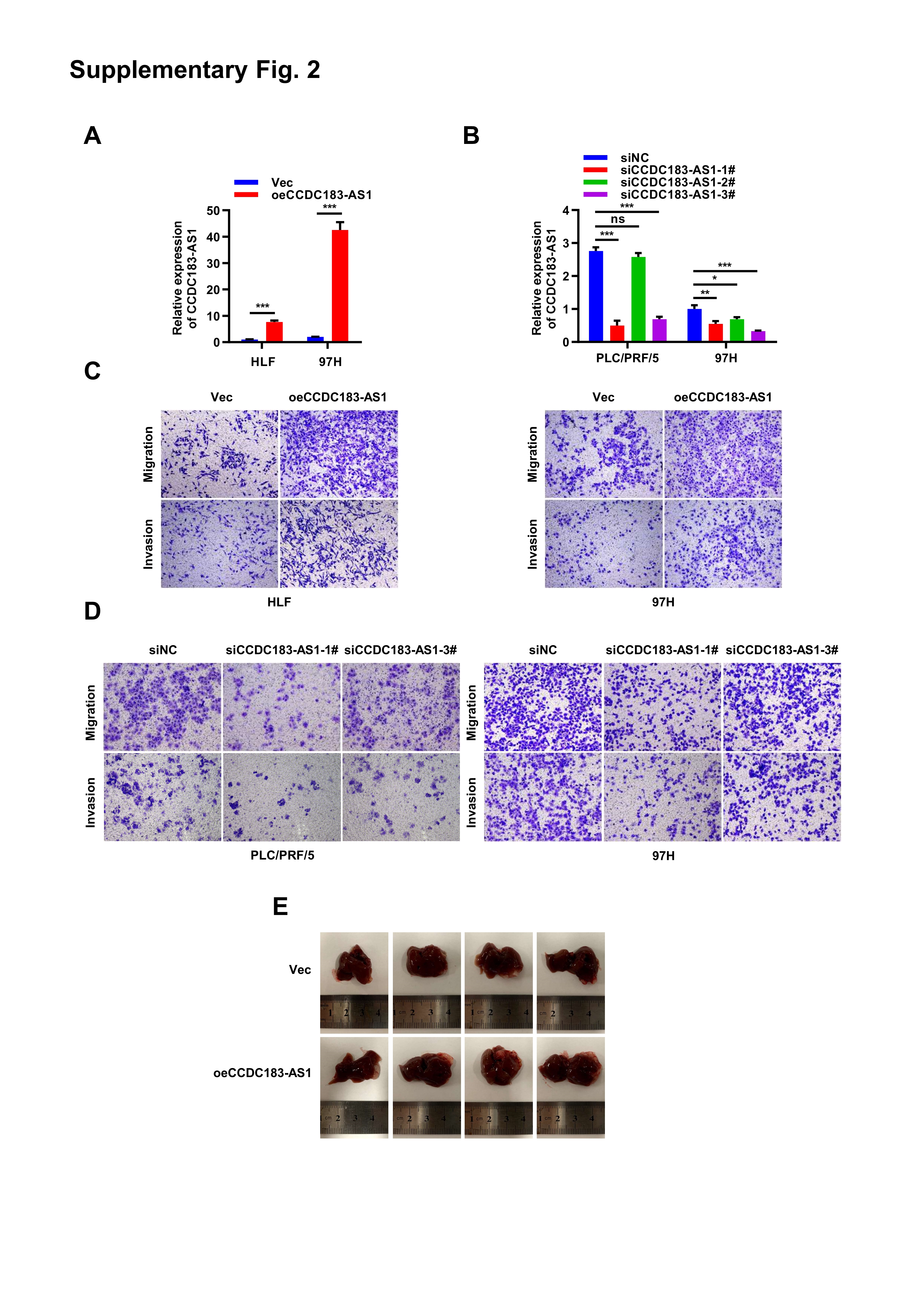

Supplement: Supplementary file 2 — Additional file 2: Supplementary Figure S2. CCDC183-AS1 promoted HCC cell proliferation and metastasis in vitro and in vivo. a-b. The overexpression (a) and knockdown (b) efficiency of CCDC183-AS1 were examined by qRT-PCR. c-d. Representative images of transwell assays in CCDC183-AS1 overexpressed (c) or knockdown (d) HCC cells. e. Representative images of the liver of CCDC183-AS1 overexpressing group and control group. Data are presented as mean ± SD. *p < 0.05, **p < 0.01, ***p < 0.001; ns, no significance. [file 13046_2021_1861_MOESM2_ESM.jpg]

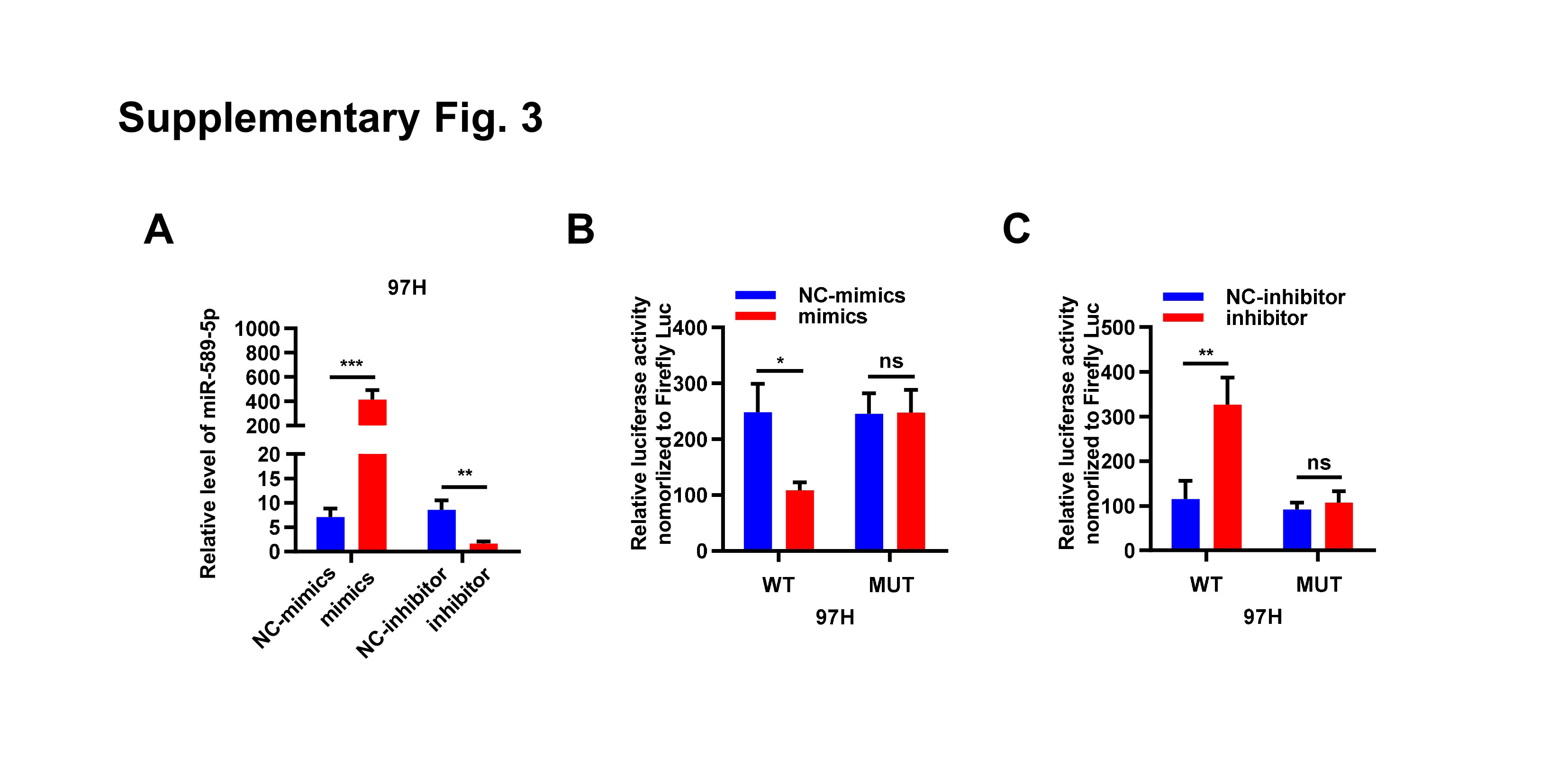

Supplement: Supplementary file 3 — Additional file 3: Supplementary Figure S3. CCDC183-AS1 acted as a ceRNA to sponge miR-589-5p in HCC cells. a. Relative expression levels of miR-589-5p were evaluated by qRT-PCR in 97H cells transfected with the miR-589-5p mimics or inhibitor, respectively. b-c. The relative luciferase activities were detected in 97H cells after co-transfection with CCDC183-AS1-WT or CCDC183-AS1-MUT and mimics, inhibitor or NC, respectively. Data are presented as mean ± SD. *p < 0.05, **p < 0.01, ***p < 0.001; ns, no significance. [file 13046_2021_1861_MOESM3_ESM.jpg]

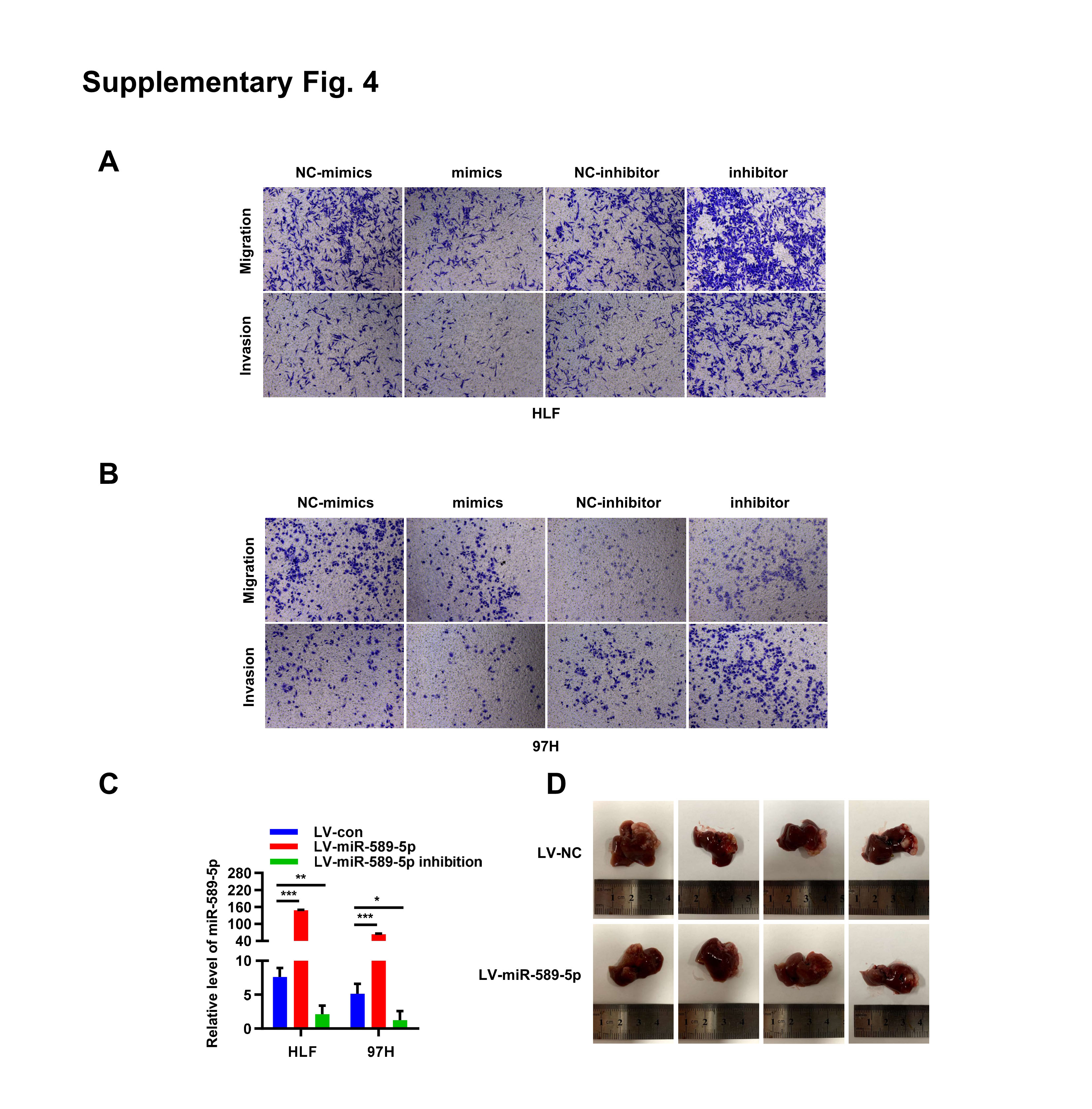

Supplement: Supplementary file 4 — Additional file 4: Supplementary Figure S4. miR-589-5p inhibited HCC cell proliferation and metastasis in vitro and in vivo. a-b. Representative images of transwell assays in HLF (a) and 97H (b) cells transfected with miR-589-5p mimics or inhibitor, respectively. c. The expression levels of miR-589-5p were examined by qRT-PCR in HLF and 97H cells with stable miR-589-5p overexpression or knockdown. d. Representative images of the liver of miR-589-5p overexpressing group and control group. Data are presented as mean ± SD. *p < 0.05, **p < 0.01, ***p < 0.001; ns, no significance. [file 13046_2021_1861_MOESM4_ESM.jpg]

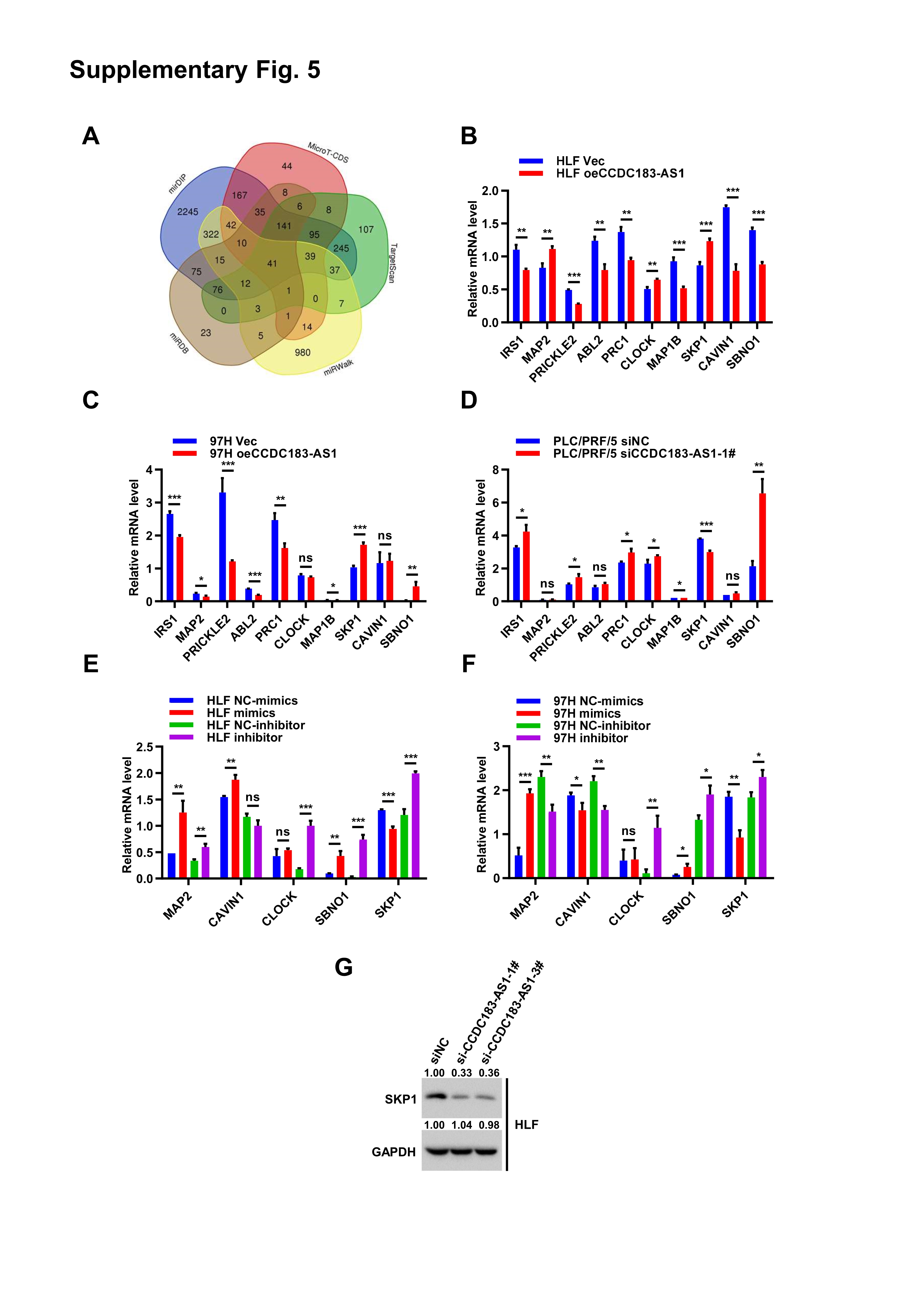

Supplement: Supplementary file 5 — Additional file 5: Supplementary Figure S5. SKP1 is a direct target of miR-589-5p. a. Venn diagrams showing the number of target genes of miR-589-5p predicted by mirDIP, MicroT-CDS, TargetScan, miRWalk and miRDB. b-d. Expression levels of candidate genes were measured by qRT-PCR after CCDC183-AS1 overexpression (b-c) or knockdown (d). e-f. qRT-PCR analysis of the expression of candidate genes in HLF (e) and 97H (f) cells when treated with miR-589-5p mimics or inhibitor. g. SKP1 protein levels in HLF cells after CCDC183-AS1 knockdown. Data are presented as mean ± SD. *p < 0.05, **p < 0.01, ***p < 0.001; ns, no significance. [file 13046_2021_1861_MOESM5_ESM.jpg]

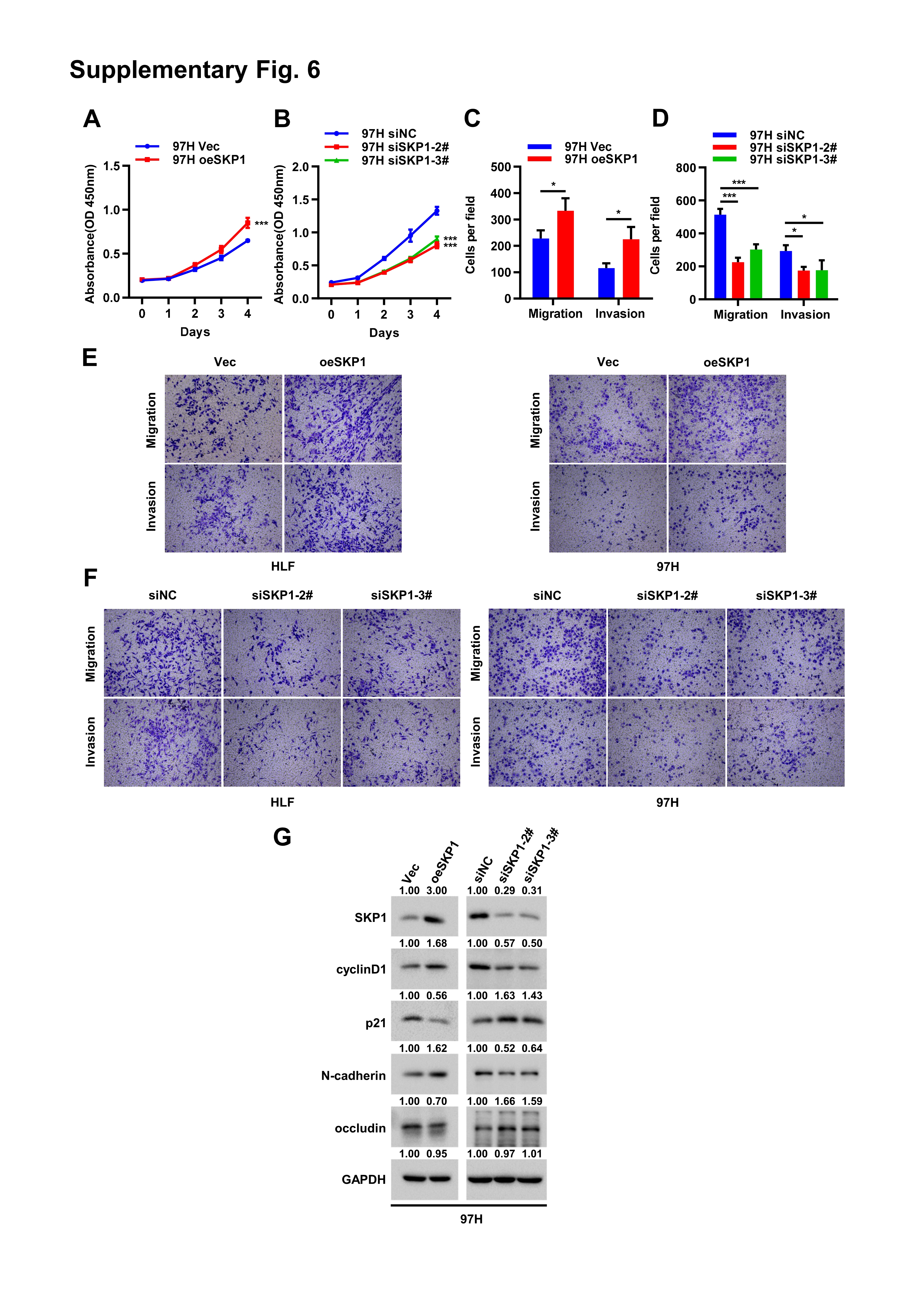

Supplement: Supplementary file 6 — Additional file 6: Supplementary Figure S6. SKP1 promoted HCC cell proliferation and metastasis. a-b. CCK-8 assays were used to evaluate 97H cells proliferation after SKP1 overexpression (a) or knockdown (b). c-d. Cellular migratory and invasive capabilities were assessed by transwell assays in 97H cells after SKP1 overexpression (c) or knockdown (d). e-f. Representative images of transwell assays in HLF and 97H cells after SKP1 overexpression (e) or knockdown (f). g. Western blot analysis to determine expression level of the cell cycle and EMT relative marker in SKP1 overexpressed and knockdown 97H cells. h. The expression levels of SKP1 in HCC and adjacent non-cancer tissues were evaluated by Western blot(n=80). Data are presented as mean ± SD. *p < 0.05, **p < 0.01, ***p < 0.001; ns, no significance. [file 13046_2021_1861_MOESM6_ESM.zip › Revised Supplementary Fig. 6-1.jpg]

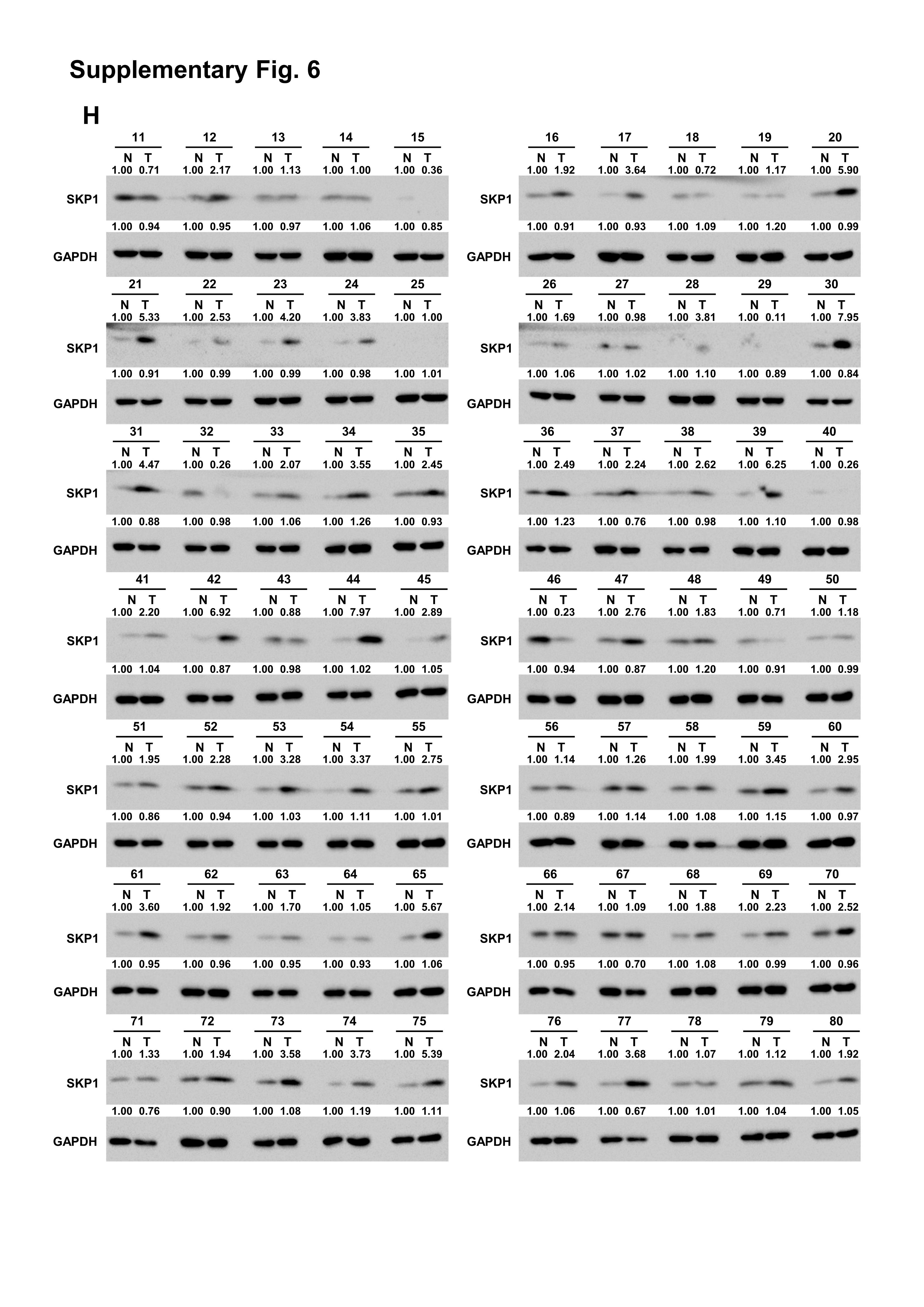

Supplement: Supplementary file 6 — Additional file 6: Supplementary Figure S6. SKP1 promoted HCC cell proliferation and metastasis. a-b. CCK-8 assays were used to evaluate 97H cells proliferation after SKP1 overexpression (a) or knockdown (b). c-d. Cellular migratory and invasive capabilities were assessed by transwell assays in 97H cells after SKP1 overexpression (c) or knockdown (d). e-f. Representative images of transwell assays in HLF and 97H cells after SKP1 overexpression (e) or knockdown (f). g. Western blot analysis to determine expression level of the cell cycle and EMT relative marker in SKP1 overexpressed and knockdown 97H cells. h. The expression levels of SKP1 in HCC and adjacent non-cancer tissues were evaluated by Western blot(n=80). Data are presented as mean ± SD. *p < 0.05, **p < 0.01, ***p < 0.001; ns, no significance. [file 13046_2021_1861_MOESM6_ESM.zip › Revised Supplementary Fig. 6-2.jpg]

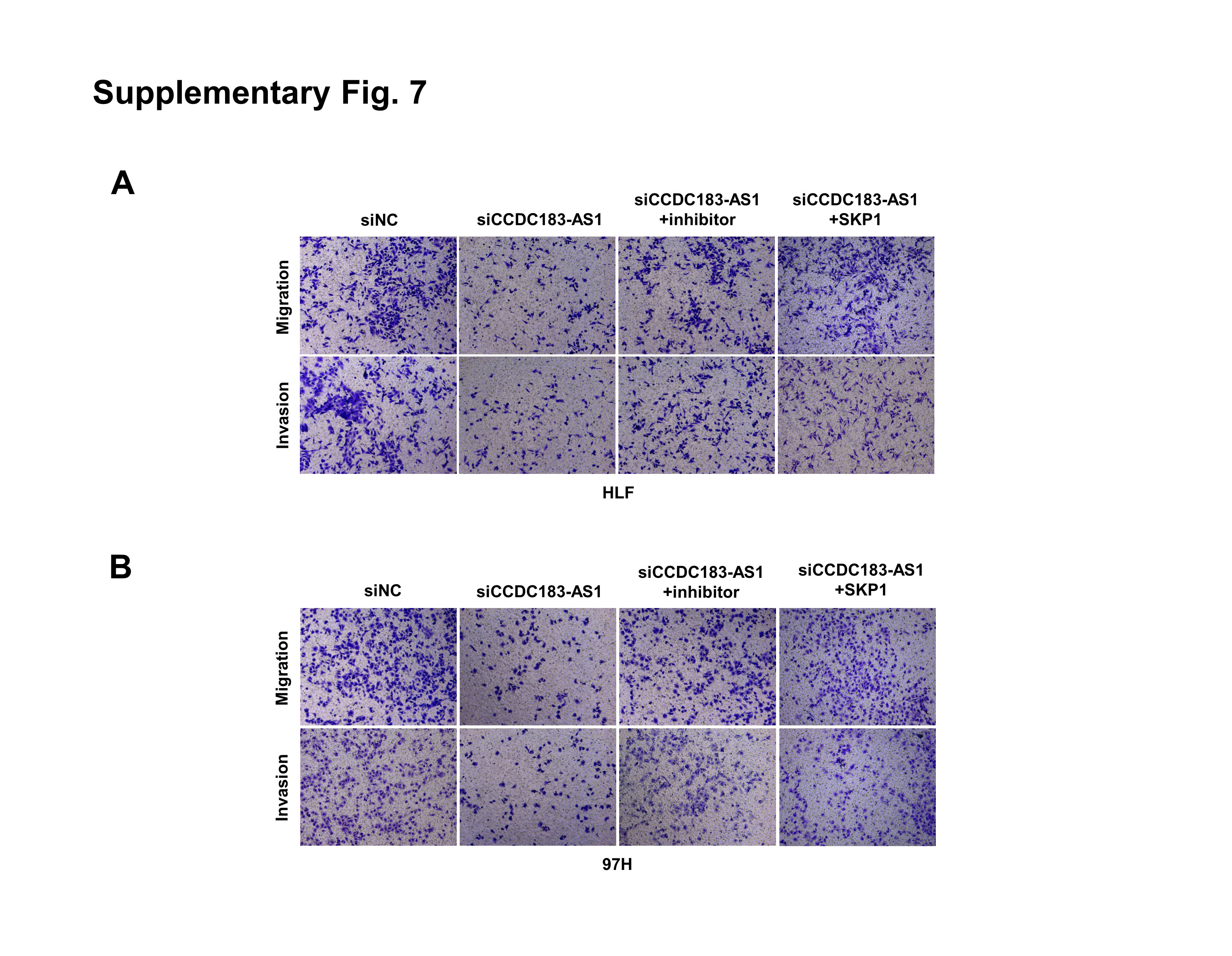

Supplement: Supplementary file 7 — Additional file 7: Supplementary Figure S7. CCDC183-AS1 promotes HCC progression through the CCDC183-AS1/miR-589-5p/SKP1 axis. a-b. Representative images of transwell assays in HLF (a) and 97H (b) cells transfected with indicated NC, siCCDC183-AS1, miR-589-5p inhibitor or SKP1, respectively. [file 13046_2021_1861_MOESM7_ESM.jpg]
